# Magnitude of nitrate turbulent diffusion in contrasting marine environments

**DOI:** 10.1038/s41598-021-97731-4

**Published:** 2021-09-22

**Authors:** Beatriz Mouriño-Carballido, José Luis Otero Ferrer, Bieito Fernández Castro, Emilio Marañón, Mariña Blazquez Maseda, Borja Aguiar-González, Paloma Chouciño, Rocío Graña, Víctor Moreira-Coello, Marina Villamaña

**Affiliations:** 1 Departamento de Bioloxía e Ecoloxía Animal, Universidade de Vigo, Vigo, Spain; 2 Ocean and Earth Science, University of Southampton, National Oceanography Centre, Southampton, SO14 3ZH UK; 3 Departamento de Física, Facultad de Ciencias del Mar, Universidad de Las Palmas de Gran Canaria, 35017 Las Palmas, Spain; 4 School of Marine Science and Policy, College of Earth, Ocean and Environment, University of Delaware, Newark, DE USA; 5 Instituto Español de Oceanografía, Xixón, Spain

**Keywords:** Marine biology, Physical oceanography

## Abstract

Difficulties to quantify ocean turbulence have limited our knowledge about the magnitude and variability of nitrate turbulent diffusion, which constitutes one of the main processes responsible for the supply of nitrogen to phytoplankton inhabiting the euphotic zone. We use an extensive dataset of microturbulence observations collected in contrasting oceanic regions, to build a model for nitrate diffusion into the euphotic zone, and obtain the first global map for the distribution of this process. A model including two predictors (surface temperature and nitrate vertical gradient) explained 50% of the variance in the nitrate diffusive flux. This model was applied to climatological data to predict nitrate diffusion in oligotrophic mid and low latitude regions. Mean nitrate diffusion (~ 20 Tmol N y^−1^) was comparable to nitrate entrainment due to seasonal mixed-layer deepening between 40°N–40ºS, and to the sum of global estimates of nitrogen fixation, fluvial fluxes and atmospheric deposition. These results indicate that nitrate diffusion represents one of the major sources of new nitrogen into the surface ocean in these regions.

## Introduction

“Follow the nitrogen”, the mantra proposed by Capone et al.^1^ for searching for life on Mars, summarizes the relevance of this element for life. By limiting both land and marine primary productivity, this element controls the flow of energy through the ecosystem, and the atmospheric CO_2_ uptake by photosynthetic organisms. Marine photosynthesis can be supported by nitrogen inputs supplied from outside the photic layer (named new production, mainly in the form of nitrate), or by nitrogen remineralized within the photic layer (regenerated production)^2^. In steady state, when large temporal and spatial scales are considered, and for fixed stoichiometry of organic matter, new production is equivalent to the amount of organic carbon that can be exported to the deep ocean, where it remains isolated over the time scale of deep ocean circulation (100 s–1000 s years)^3^. The export efficiency of organic carbon production in the ocean can be characterized in terms of the f-ratio, defined as the ratio of new production to the sum of new plus regenerated production (total production)^4^.

Nitrate turbulent diffusion has been traditionally considered one of the main mechanisms by which nitrogen is supplied to phytoplankton, particularly in permanently stratified ocean environments such as tropical and subtropical regions^5^, and in temperate areas during summer stratification^6,7^. Quantifying nitrate diffusion requires estimates of vertical turbulent diffusivity (Kz), which can be derived from observations of microstructure, tracer release experiments and acoustic measurements of flow velocities^8^. These experimental approximations present methodological difficulties that have historically limited our knowledge about the magnitude and variability of turbulent mixing in the ocean. Alternatively, constant values of Kz have been used to estimate nitrate turbulent diffusion^9^. However, the increased availability of commercial microturbulence profilers has enlarged the number of observations, which have revealed a large temporal and spatial variability of Kz in the upper layer of the ocean^10–17^.

The limited number of studies that have simultaneously quantified the relevance of different new nitrogen supply mechanisms, by using observations at different spatial scales, have reported contradictory results about the role of nitrate diffusion. With measurements across the tropical and subtropical Atlantic, Pacific and Indian oceans, Fernández-Castro et al.^18^ showed that nitrate diffusion (171 ± 190 mmolm^−2^ d^−1^) clearly dominated over N_2_ fixation (9.0 ± 9.4 mmolm^−2^ d^−1^) at the time of sampling. When comparing with atmospheric nitrogen deposition and biological nitrogen fixation across a longitudinal section in the Mediterranean Sea, the contribution of nitrate diffusion to new production ranged from 0.1 to 25%^19^. In the northeast subtropical Atlantic, Painter et al.^20^ reported nitrate diffusive fluxes comparable to N_2_ fixation rates. Finally, according to Caffin et al.^21^ N_2_ fixation was the major source of new nitrogen (> 90%), compared to nitrate diffusion and atmospheric deposition, in the western tropical South Pacific. The bulk estimates of seawater nutrient concentration have been also frequently used as a proxy for nutrient availability in the euphotic zone^22,23^. However, nitrate concentrations and actual nitrate supply into the euphotic zone can be disconnected in near-steady-state systems, such as the subtropical gyres, where nitrate diffusion is slow, and nitrate concentrations are kept close to the detection limit due to phytoplankton uptake^24,25^.

We used a large dataset of microturbulence observations and a multivariable fractional polynomial method to investigate the relationship between nitrate turbulent diffusion and environmental variables that are routinely measured during oceanographic cruises. The observed relationship was used to build the first large-scale map of the distribution of the supply of nitrate into the euphotic zone through turbulent diffusion.

## Results and discussion

### Characteristics of the sampled regions

**Figure 1:**
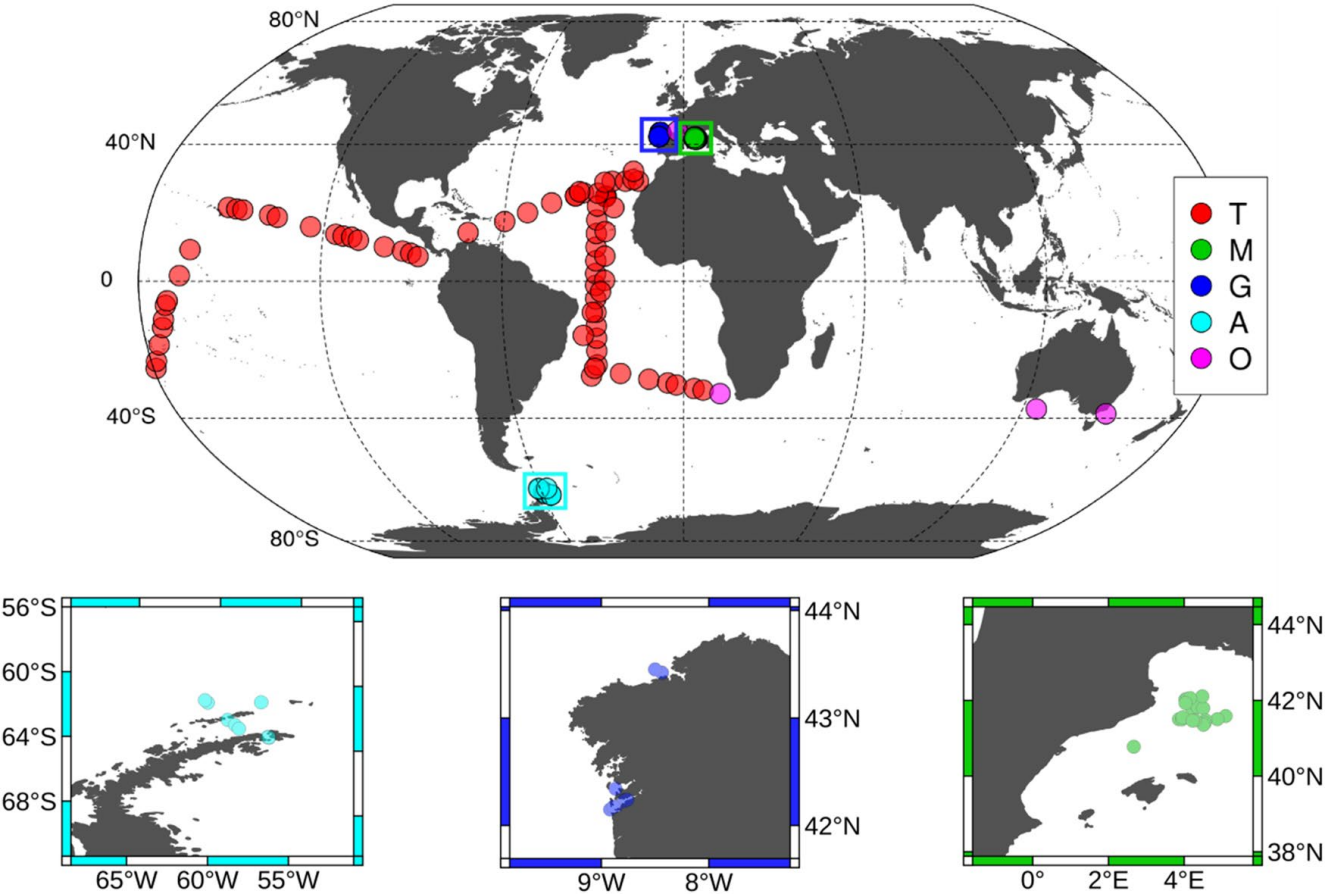
Location of the stations used in this study. Stations sampled in tropical and subtropical regions (T) are shown in red, the Mediterranean Sea (M) in green, the Galician coastal upwelling (G) in dark blue, the Antarctic Peninsula (A) in light blue and other regions (O) in pink. Small panels provide details about those stations sampled in the Antarctic Peninsula (light blue), the Galician coastal upwelling (dark blue) and the Mediterranean Sea (green). Figure was generated by using Matlab R2018b (https://es.mathworks.com).

This study includes 181 stations collected between October 2006 and December 2015 mainly in four contrasting marine environments: tropical and subtropical Atlantic and Pacific oceans (T), Northwestern Mediterranean Sea (M), Galician coastal upwelling (G), and Antarctic Peninsula (A) (see Table 1 and Fig. 1). Three cruises (CARPOS Oct–Nov 2006, TRYNITROP Apr–May 2008 and MALASPINA Dic 2010–July 2011) sampled 73 stations, mainly located in the tropical and subtropical Atlantic and Pacific Oceans. Four cruises carried out in the Northwestern Mediterranean Sea (PERFILM Jun–Jul 2009, FAMOSO1 Mar 2009, FAMOSO2 Apr–May 2009, FAMOSO3 Sep 2009) sampled 34 stations during four contrasting hydrographic conditions, covering from winter mixing to summer stratification. In the Galician coastal upwelling 37 stations were sampled during seven cruises, spanning all seasons and representative conditions: HERCULES2 Sep 2011, HERCULES3 Jul 2012, DISTRAL-REIMAGE Feb 2012–Jan 2013, ASIMUTH Jun 2013, STRAMIX Aug 2013, CHAOS Aug 2013, and NICANOR Feb 2014–Dec 2015 cruises. Most stations carried out in the Galician coastal upwelling were conducted inside three different Rías (Ría de Vigo, Ría de Pontevedra and Ría de A Coruña). The Rías are coastal bays influenced by seasonal wind-driven coastal upwelling which bring cold, nutrient-rich North Atlantic Central water to the surface^26^. In this region all stations considered were deeper than 40 m. One cruise sampled 10 stations located in the Antarctic Peninsula during the austral summer (COUPLING Jan-2010). Finally, the PERFILC cruise sampled 27 stations in the Bay of Biscay in July 2008. hHydrographic and microstructure turbulence profiles collected during both cruises were averaged arithmetically to obtain mean profiles which were assigned to the mean geographical location of all the stations conducted during each cruise (see Methods). Additional information about the sampling design of these cruises is described in Aranguren-Gassis et al.^27^, Mouriño-Carballido et al.^28^, Fernández-Castro et al.^18^, Mouriño-Carballido et al.^24^, Cermeño et al.^29^, Teira et al.^30^, Villamaña et al.^15^, Díaz et al.^31^, and Moreira-Coello et al.^32^.

**Table 1:** Details of the data included in this study.

Domain	N	Cruise	Vessel	Date (dd/mm/yy)	Duration	Depth
NE Atlantic (T)	8	CARPOS	*Hespérides*	14/10/06–22/11/06	49 ± 25	137 ± 14
Atlantic (T)	18	TRYNITROP	*Hespérides*	14/04/08–02/05/08	32 ± 12	219 ± 19
Atlantic and Pacific (T)	47	MALASPINA	*Hespérides*	19/12/10–10/07/11	27 ± 9	234 ± 23
NW Mediterranean (M)	15	PERFILM	*García del Cid*	27/06/09–1/07/09	51 ± 22	201 ± 12
Liguro-Provençal Basin (M)	6	FAMOSO1	*Sarmiento de Gamboa*	14/3/09–22/3/09	66 ± 5	259 ± 38
Liguro-Provençal Basin (M)	10	FAMOSO2	*Sarmiento de Gamboa*	30/4/09–13/05/09	94 ± 4	273 ± 2
Liguro-Provençal Basin (M)	3	FAMOSO3	*Sarmiento de Gamboa*	16/09/09–20/09/09	133 ± 3	323 ± 24
Ría de A Coruña (G)	2	HERCULES2	*Lura*	28/09/11–29/09/11	11 ± 8	77 ± 11
Ría de A Coruña (G)	5	HERCULES3	*Lura*	16/07/12–20/07/12	8 ± 4	67 ± 24
Ría de Vigo (G)	10	DISTRAL & REIMAGE	*Mytilus*	14/02/12–24/1/2013	110 ± 76	38 ± 2
Ría de Vigo (G)	1	STRAMIX	*Mytilus*	5–6/8/2013	1614	38 ± 2
Ría de Vigo (G)	2	CHAOS	*Mytilus*	20/08/13–27/08/13	1515 ± 6	41 ± 1
Ría de A Coruña (G)	13	NICANOR	*Lura*	27/02/14–17/12/15	30 ± 5	62 ± 3
Rías de Vigo & Pontevedra (G)	4	ASIMUTH	*Ramón Margalef*	17/06/13–21/06/13	16 ± 7	54 ± 17
South Shetland Islands (A)	10	COUPLING	*Hespérides*	8/1/10 – 21/1/10	57 ± 5	442 ± 41
Bay of Biscay (O)	27	PERFILC	*García del Cid*	18/7/08–24/7/08	35 ± 13	226 ± 39

Domain refers to tropical and subtropical Atlantic and Pacific oceans (T), Northwestern Mediterranean Sea (M), Galician coastal upwelling (G), Antarctic Peninsula (A) and other regions (O). N indicates the number of stations sampled during each cruise. Duration (mean ± standard deviation, in minutes) is the time used for the turbulence profiler deployment at each station. Depth (mean ± standard deviation, in meters) is the maximum depth reached by the microstructure profiler. During the Malaspina expedition only 44 out of 47 stations were considered as tropical and subtropical.

**Figure 2:**
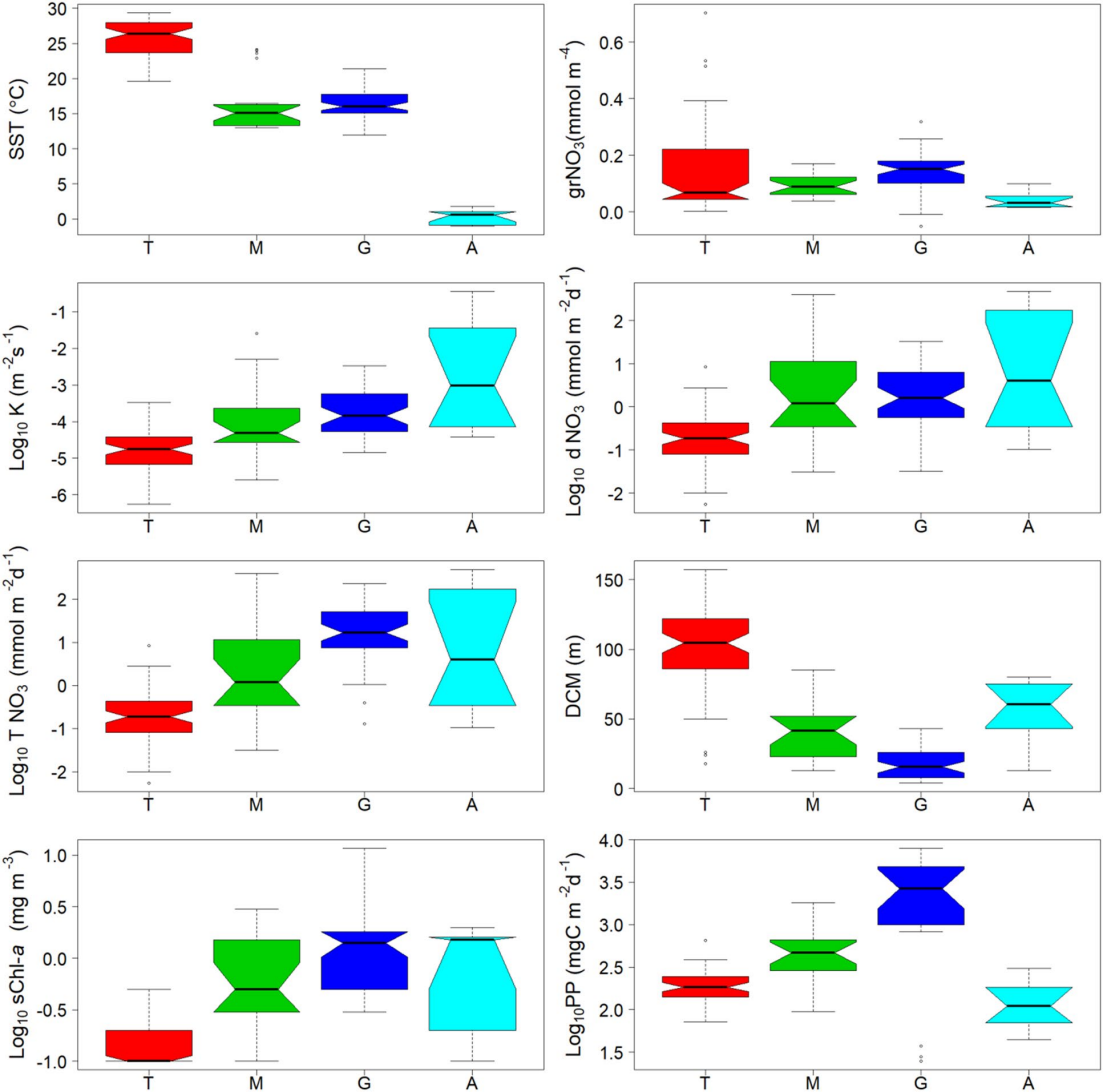
Box-and-whisker plots of Sea surface temperature (SST), vertical nitrate gradient (grNO_3_), vertical diffusivity (K), nitrate diffusive flux (dNO_3_), total nitrate supply (including vertical diffusion plus advection, TNO_3_), depth of the deep chlorophyll maximum (DCM), surface chlorophyll-a (sChl-a), and photic layer depth-integrated net primary production (PP) computed for the tropical and subtropical regions (T), the Northwestern Mediterranean (M), the Galician coastal upwelling (G) and the Antarctic (A). On each box, the central mark indicates the median, the notches the 95% confidence interval for the median, and the bottom and top edges of the box indicate the 25th and 75th percentiles, respectively. The whiskers extend to the most extreme data points not considered outliers, and the outliers are plotted individually using white circles.

This database covered a wide environmental gradient from oligotrophic to eutrophic conditions. Temporal variability due to seasonal changes in environmental forcing was partially captured in some regions (Northwestern Mediterranean Sea and Galician coastal upwelling) but mainly missed in others (tropical and subtropical Atlantic and Pacific oceans and the Antarctic Peninsula. The 70 stations sampled in the tropical and subtropical Atlantic and Pacific oceans were, on average, characterized by relatively warm surface temperature (26 ± 3 °C, mean ± SD), weak vertical diffusivity (0.03 ± 0.05 × 10^–3^ m^2^ s^−1^) and low nitrate diffusive supply (0.4 ± 1 mmol N m^−2^ d^−1^) (Fig. 2 and Table 2). Accordingly, surface chlorophyll-a and photic layer depth-integrated net primary production were low (0.2 ± 0.1 mg m^−3^ and 202 ± 98 mg C m^−2^ d^−1^, respectively). The Mediterranean Sea, was characterized by cooler surface waters (16 ± 4 °C), and relatively large diffusivity (2 ± 6 × 10^–3^ m^2^ s^−1^) and nitrate diffusive supply (39 ± 110 mmol N m^−2^ d^−1^). Surface chlorophyll-a and net primary production took intermediate values (0.9 ± 0.9 mg m^−3^ and 581 ± 454 mg C m^−2^ d^−1^, respectively). The stations sampled in the Galician coastal were characterized, on average, by relatively cold surface water (16 ± 2 °C), and intermediate diffusivity (0.4 ± 0.5 × 10^–3^ m^2^ s^−1^) and nitrate diffusive supply (5.3 ± 7.1 mmol N m^−2^ d^−1^). However, due to the influence of coastal upwelling the total nitrate supply (including both diffusive and advective processes) was significantly higher (31 ± 42 mmol N m^−2^ d^−1^). As the result of the input of new nitrogen by both processes this system was characterized by high values of surface chlorophyll-a and net primary production (2.5 ± 2.9 mg m^−3^ and 2950 ± 2330 mg C m^−2^ d^−1^, respectively). Finally, the 10 stations sampled in the Antarctic Peninsula were characterized by cold surface water (0.1 ± 1.0 °C), high diffusivity (66 ± 126 × 10^–3^ m^2^ s^−1^) and high nitrate diffusive supply (102 ± 165 mmol N m^−2^ d^−1^). However, due to the limitation of control factors other than nitrate^30^, these regions exhibited intermediate values of surface chlorophyll-a and relatively low net primary production (1.1 ± 0.8 mg m^−3^ and 153 ± 136 mg C m^−2^ d^−1^, respectively).

**Table 2:** Mean ± standard deviation of selected variables.

Variables (units)	T	M	G	A	K-W p_value_	Post hoc Bonferroni
SST (ºC)	26 ± 3	16 ± 4	16 ± 2	0.1 ± 1.0	< 0.001	T > M,G,A; G > A
SSS (PSU)	36 ± 1	38 ± 0.1	35 ± 0.4	34 ± 0.9	< 0.001	M > T,G > A
maxN^2^ × 10^–4^ (s^−2^)	7 ± 7	3 ± 5	11 ± 12	4 ± 10	< 0.001	G > T > M,A
dmaxN^2^ (m)	74 ± 43	35 ± 17	16 ± 14	94 ± 92	< 0.001	T > M > G; A > G
avrN^2^ × 10^–4^ (s^−2^)	2.1 ± 3	0.7 ± 0.5	1.9 ± 1.4	0.3 ± 0.6	< 0.001	G > T,M,A; T > A
MLD (m)	53 ± 23	32 ± 29	15 ± 13	174 ± 198	< 0.001	T,A > M,G
K × 10^–3^ (m^2^ s^−1^)	0.03 ± 0.05	2 ± 6	0.4 ± 0.5	66 ± 126	< 0.001	M,G,A > T
nitraD (m)	109 ± 59	9 ± 12	9 ± 16	5 ± 0	< 0.001	T > M,G,A
grNO_3_ (µmol m^−4^)	142 ± 149	92 ± 37	124 ± 86	42 ± 29	< 0.05	T,G > A
sNO_3_ (mM)	0.5 ± 1.2	2.3 ± 1.7	1.7 ± 1.7	31 ± 4	< 0.001	M,G,A > T; A > G
dNO_3_ (mmolN m^−2^ d^−1^)	0.4 ± 1.0	39 ± 110	5.3 ± 7.1	102 ± 165	< 0.001	M,G,A > T
TNO_3_ (mmolN m^−2^ d^−1^)	0.4 ± 1.0	39 ± 110	31 ± 42	102 ± 165	< 0.001	M,G,A > T
DCM (m)	100 ± 32	41 ± 20	16 ± 11	54 ± 24	< 0.001	T > M > G; A > G
maxCHL-a (mg m^−3^)	0.5 ± 0.2	1.1 ± 0.9	4.3 ± 3.7	1.3 ± 0.8	< 0.001	G > T,M,A
sCHL-a (mg m^−3^)	0.2 ± 0.1	0.9 ± 0.9	2.5 ± 2.9	1.1 ± 0.8	< 0.001	M,G,A > T
PP (mgC m^−2^ d^−1^)	202 ± 98	581 ± 454	2950 ± 2330	153 ± 136	< 0.001	G > T,A; M > T

Sea surface temperature (SST), sea surface salinity (SSS), vertical stratification in the pycnocline (maxN^2^), depth of the pycnocline (dmaxN^2^), average stratification in the nitracline (avrN^2^), depth of the mixed layer (MLD), vertical turbulent diffusivity (K), depth of the nitracline (nitraD), nitrate gradient across the nitracline (grNO_3_), surface nitrate concentration (sNO_3_), diffusive nitrate supply (dNO_3_), total nitrate supply (including diffusive and advective fluxes, TNO_3_), depth (DCM) and value (maxCHL-a) of the maximum chlorophyll-a, surface chlorophyll-a (sCHL-a), and photic layer depth-integrated net primary production (PP), computed for the tropical and subtropical regions (T), the Mediterranean Sea (M), the Galician coastal upwelling (G), and the Antarctic Peninsula (A). A nonparametric one-way ANOVA (Kruskal–Wallis, KW) was performed to test the null hypothesis that independent groups come from the same distribution. The Bonferroni multiple comparison test was applied a posteriori to analyze the differences between every pair of groups.

### Multivariable fractional polynomial models

At each station a set of twelve physical, chemical and biological variables regularly obtained during oceanographic cruises was selected as potential predictors of the nitrate supply by turbulent diffusion: sea surface temperature (SST), sea surface salinity (SSS), vertical stratification in the pycnocline (maxN^2^, defined as the maximum value of the squared buoyancy frequency), depth of the pycnocline (dmaxN^2^, depth of the maximum value of the squared buoyancy frequency), average stratification in the nitracline (avrN^2^, mean squared buoyancy frequency in this layer), depth of the mixed layer (MLD, calculated using a density difference criterion of 0.1 kg/m^3^ with respect to the surface value), depth of the nitracline (nitraD, the shallowest depth were nitrate concentration was equal to 1 mmol m^-3^), nitrate gradient across the nitracline (grNO_3_), surface nitrate concentration (sNO_3_), depth (DCM) and value (maxCHL-a) of the chlorophyll-a maximum, and surface chlorophyll-a (sCHL-a).

**Figure 3:**
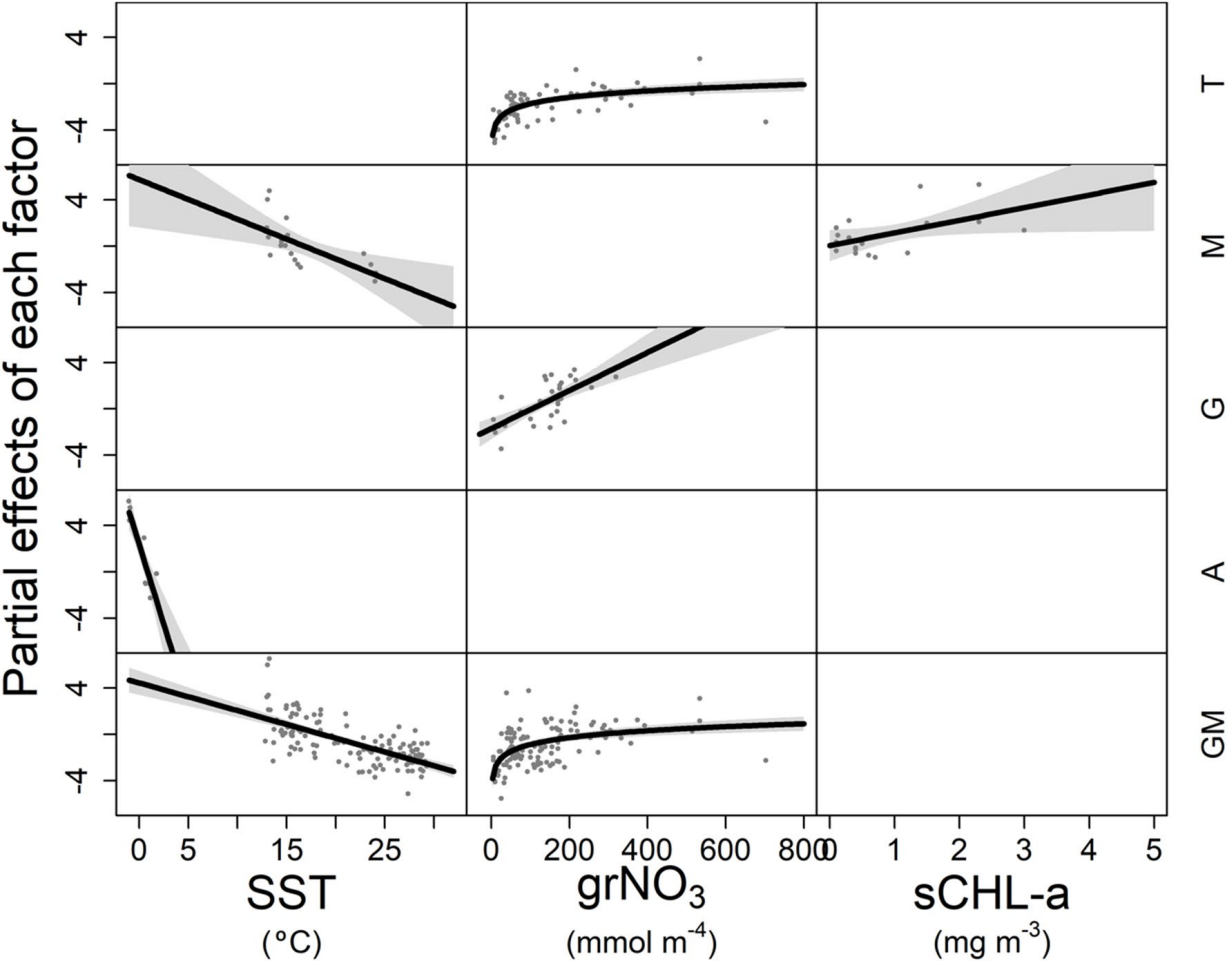
Partial effects and residuals of nitrate diffusive flux as a function of predictors. The response variable was computed as a smooth function of sea surface temperature (SST), vertical nitrate gradient (grNO_3_) and surface chlorophyll-a (sCHL-a) for tropical and subtropical regions (T), the Northwest Mediterranean (M), the Galician coastal upwelling (G), the Antarctic Peninsula (A), and for the complete dataset except those stations collected in the Antarctic Peninsula (GM). All terms were centered in zero. Relationships where p-value < 0.05 are indicated as solid black lines, whereas no significant relationships are indicated as empty pannels. Intercepts and parameters are indicated in Table 3.

In order to exclude cross-correlation between predictors and consider the possibility of non-linear relationships, a Multivariable Fractional Polynomial (MFP) method^33^ was used to select the predictors in the four contrasting environments and the complete dataset excluding those stations located in the Antarctic Peninsula (GM) (Table 3), where surface temperature was below 15 °C (Fig. 3). In a first exploratory step we allowed the models to introduce any of the twelve potential predictors (exploratory models). In tropical and subtropical regions a model including four predictors (grNO_3_, SSS, avrN^2^ and sNO_3_) explained 66% of the variance (Adj-R^2^) in nitrate diffusive fluxes, whereas in the Mediterranean and the Galician coastal upwelling two predictors (SST and sCHL-a, and grNO_3_ and MLD; respectively) explained more than 55% of the variance. In the Antarctic Peninsula, a model including only SST explained 75% of the variance. Finally, six predictors were selected for the GM global model (grNO_3_, sNO_3_, avrN^2^, SST, dmaxN^2^, maxN^2^; Adj-R^2^ = 0.63).

**Table 3:** Regression equations obtained by the MFP method in each domain (D) by using exploratory and 3-variable models.

D	Adj-R^2^	Variable	Functional form	Coefficient	Std Error	p_value_
T	0.66	Intercept		− 23.28	3.416	p < 0.001
		grNO_3_	log(grNO_3_/100)^0.5^	1.499	0.191	p < 0.001
		SSS	SSS/100	49.81	9.141	p < 0.001
		avrN^2^	(avrN^2^/10^–4^)^2^	0.032	0.012	p < 0.01
			(avrN^2^/10^–4^)^3^	− 0.002	0.001	p < 0.01
		sNO_3_	log(sNO_3_ + 0.1)	− 1.136	0.175	p < 0.001
			(sNO_3_ + 0.1)	0.783	0.134	p < 0.001
	0.27	Intercept		1.307	1.285	
		grNO_3_	log(grNO_3_/100)	0.590	0.118	p < 0.001
M	0.63	Intercept		5.210	2.300	p < 0.05
		SST	SST/10	− 3.420	1.188	p < 0.05
		sCHL-a	sCHL-a	1.090	0.538	p < 0.1
G	0.56	Intercept		− 2.772	0.624	p < 0.001
		grNO_3_	grNO_3_/100	1.940	0.305	p < 0.001
		MLD	MLD/10	0.433	0.181	p < 0.05
	0.49	Intercept		− 1.702	0.468	p < 0.001
		grNO_3_	grNO_3_/100	1.641	0.299	p < 0.001
A	0.75	Intercept		5.410	0.842	p < 0.001
		SST	SST + 1.1	− 2.751	0.514	p < 0.001
GM	0.63	Intercept		2.148	0.759	p < 0.01
		grNO_3_	(grNO_3_/100)^0.5^	1.738	0.300	p < 0.001
		sNO_3_	sNO_3_ + 0.1	0.246	0.087	p < 0.01
		avrN^2^	(avrN^2^/10^–4^)	− 0.255	0.062	p < 0.001
		SST	SST/10	− 2.370	0.328	p < 0.001
		dmaxN^2^	(dmaxN^2^/100)^2^	1.482	0.491	p < 0.01
			(dmaxN^2^/100)^2^*log(dmax N^2^/100)	− 2.120	0.647	p < 0.01
		maxN^2^	log(maxN^2^/0.001)	0.909	0.272	p < 0.01
			log(maxN^2^/0.001)^2^	0.372	0.091	p < 0.001
	0.50	Intercept		2.851	0.576	p < 0.001
		SST	SST/10	− 2.471	0.243	p < 0.001
		grNO_3_	log(grNO_3_/100)^0.5^	1.636	0.274	p < 0.001

T indicates tropical and subtropical regions, M Mediterranean Sea, G Galician coastal upwelling, A Antarctic Peninsula, and GM global model built with the complete dataset except those collected in the Antarctic Peninsula. Exploratory models used all the predictors, and 3-variable models only used sea surface temperature (SST), surface chlorophyll-a (sCHL-a) or vertical nitrate gradient (grNO_3_). Adj-R^2^ is adjusted squared coefficient of determination, and Std Error standard error. The number of stations used to build each model is indicated in Table 1.

**Figure 4:**
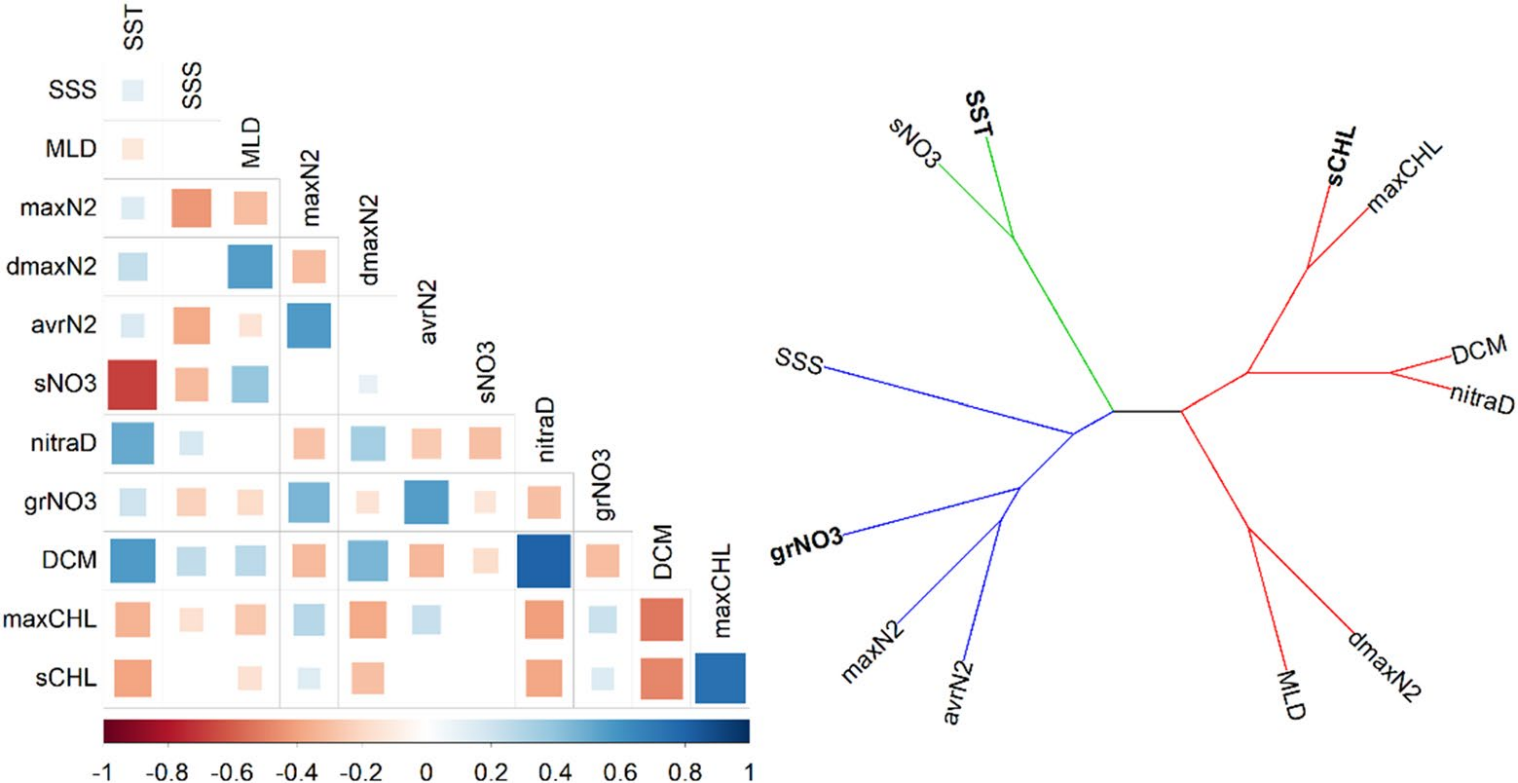
Correlogram and dendrogram. Left: Correlogram of predictive variables where statistically significant correlations (statistical confidence level of 95%) are shown. The test was based on Pearson’s product moment correlation, and alternative hypothesis include both sides. A Bonferroni correction was applied to p-values to control Type-I error. The color indicates the sign and the size indicate the magnitude of the correlation. Right: Dendrogram created using the correlations between predictive variables. Dissimilarity was established as 1- |correlation|. Bold letters indicate the predictors selected to be used in the simplified 3-variable models.

In order to build more simple models by limiting the number of potential predictors, we investigated the correlation between predictors by using a correlogram (Fig. 4). The cluster analysis created using a dendrogram based on the correlation matrix between the predictors showed that variables could be clustered into three groups. Among these clusters we selected three variables (SST, grNO_3_, and sCHL-a) to build the 3-variable models. The three selected predictors were chosen because they are routinely measured during oceanographic cruises, and also derived from satellite observations or available on global oceanographic databases. This was not necessary for the Mediterranean and the Antarctic, where the exploratory models already selected a combination of these predictors. For tropical and subtropical regions the algorithm retained one variable (grNO_3_), which explained about half of the variance (27%) compared to the model with all the potential predictors included. In the Galician coastal upwelling only grNO_3_ was retained, but the decrease in explained variance was smaller (Adj-R^2^ = 0.49). The global model maintained its accuracy very close (Adj-R^2^ = 0.50) after decreasing the number of predictors to two variables (SST and grNO_3_). Several factors could explain the variability in the variance explained by the different regional 3-variable models. First, the dataset collected in the Antarctic and, specially, in the Mediterranean included contrasting hydrographic conditions and a wider range of nitrate diffusive fluxes than the other regions (Fig. 2). Second, in the Galician upwelling nitrate turbulent diffusion represents, on average, a minor source of new nitrogen into the euphotic zone compared to the nitrate flux driven by vertical advection of deeper waters through upwelling^32^. Moreover, it is noteworthy that the explained variance in tropical and subtropical regions decreased significantly from the exploratory (66%) to the 3-variable models (27%), whereas the reduction in the number of predictors had a limited effect in the other regions. This could be related to the fact that the observations included in our database barely cover seasonal changes in hydrographic conditions in this oceanic domain.

The dependence of nitrate diffusion on the different explanatory variables could be interpreted in terms of the underlying physical and ecological processes. All models in which SST was included exhibited negative linear relationships between nitrate diffusive fluxes and this variable (Fig. 3). The vertical nitrate gradient exhibited a positive linear correlation with nitrate diffusive flux in the model built for the Galician coastal upwelling. For tropical and subtropical regions and the global model, the relationship with the vertical nitrate gradient was also positive but logarithmic. Finally, surface chlorophyll-a showed a linear or nearly linear positive relationship with nitrate diffusive fluxes in the Mediterranean. Sea surface temperature is related to vertical stratification, as stratification usually increases with higher surface temperature. As expected, a statistically significant positive correlation was found between the two variables in our dataset (Fig. 4). In turn, assuming no systematic compensatory changes by increased dissipation rates of turbulent kinetic energy or enhanced vertical nitrate gradient, vertical diffusivity, and therefore nitrate diffusive fluxes, decreases when stratification increases (see Methods). This could explain the negative relationship observed between surface temperature and nitrate diffusive flux for all models where this predictor was included. The relationship between the vertical nitrate gradient and nitrate diffusive flux was positive, as this term is directly involved in the calculation of diffusive fluxes (see Methods). Finally, when included, the relationship between surface chlorophyll-a and nitrate diffusive fluxes was positive, as this predictor is an estimator of phytoplankton biomass, and therefore reflects the response of these organisms to nitrate availability.

### Inferred global distribution of nitrate diffusion into the photic zone

**Figure 5:**
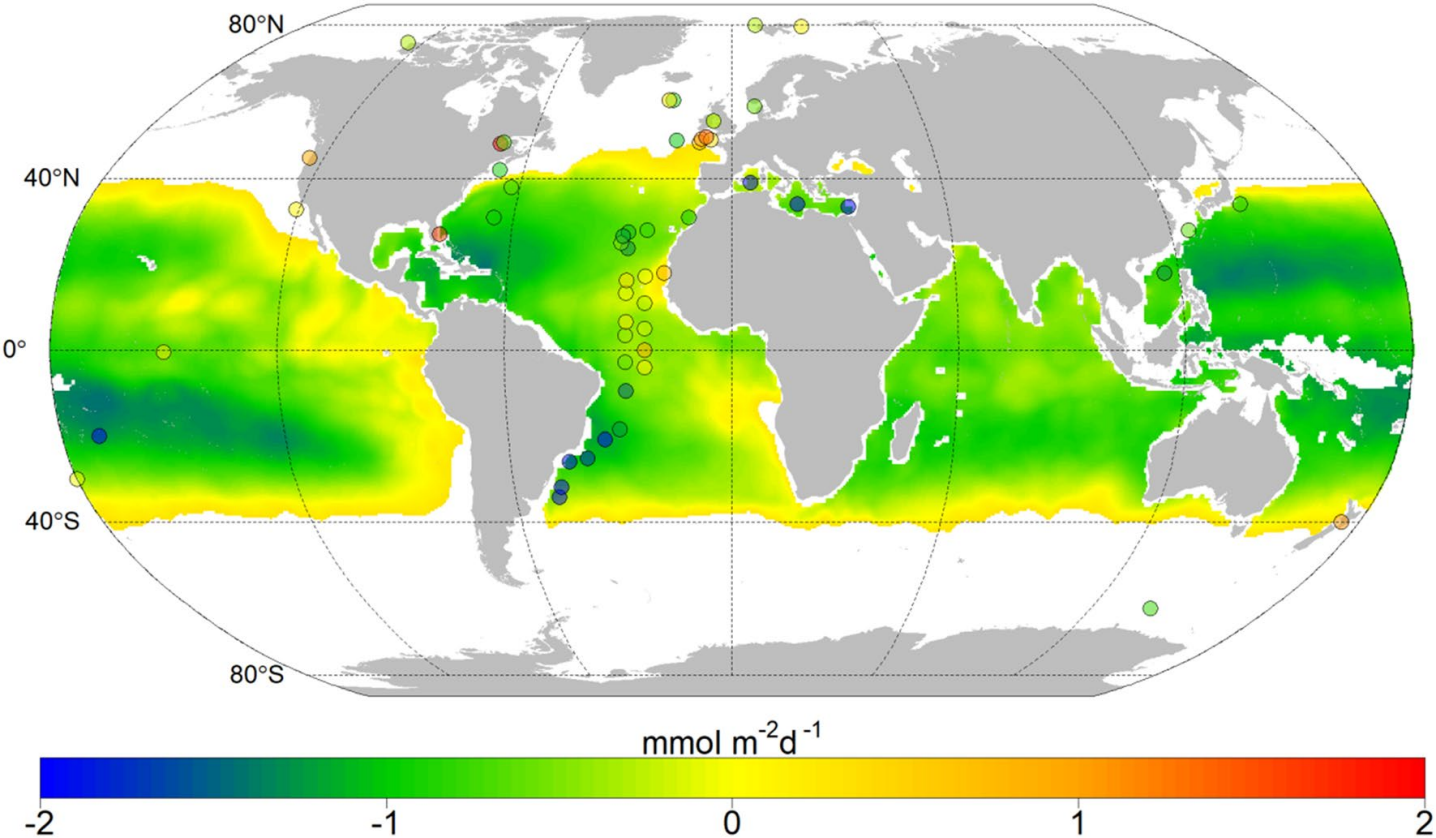
Global distribution of the decimal logarithm of nitrate diffusive fluxes derived by using the GM model specified in Table 3. The colored dots represent nitrate diffusive fluxes previously reported in the literature and described in Table 5. The white color indicates those regions where data were outside of the range covered by the dataset used to build the prediction model. Figure was generated by using RStudio (https://www.rstudio.com) (Version 1.1.456).

A large-scale estimate of the distribution of the supply of nitrate into the photic zone by vertical turbulent diffusion was computed by using the global 3-variable model derived from the complete dataset, excluding those stations collected in the Antarctic Peninsula, and including as predictors SST and grNO_3_ from the the World Ocean Atlas 2009 (WOA09,. http://www.nodc.noaa.gov/) (Fig. 5). As expected based on the regional differences observed in our estimates of nitrate diffusive fluxes derived from observations (Fig. 2), regional patterns of this distribution revealed an increase in the magnitude of nitrate supply from open ocean tropical and subtropical regions to upwelling, coastal and temperate regions. Mean nitrate fluxes for the biogeographical provinces defined in the tropical and subtropical Atlantic ocean^34^, where most stations were collected, were 0.58 ± 0.33 mmolN m^−2^ d^−1^ for NASE (NE Atlantic Subtropical Gyral), 0.19 ± 0.10 mmolN m^−2^ d^−1^ for NASW (NW Atlantic Subtropical Gyral), 0.26 ± 0.25 mmolN m^−2^ d^−1^ for NATR (NorthAtlantic Tropical Gyral), 0.56 ± 0.28 mmolN m^−2^ d^−1^ for ETRA (Eastern tropical Atlantic), 0.33 ± 0.12 mmolN m^−2^ d^−1^ for WTRA (Western Tropical Atlantic), and 0.59 ± 0.56 mmolN m^−2^ d^−1^ for SATL (South Atlantic Gyral). A detailed list of mean nitrate fluxes for main open ocean biogeographical provinces defined by Longhurst^34^ between 40° N–40° S is included in Table 4. Because SST and grNO3 data obtained from WOA09 include observations collected at different seasons, mean nitrate fluxes computed for the biogeographical provinces can be considered as spatio-temporal means.

**Table 4:** Variables for selected biogeographical provinces.

Biogeographical province	Ocean	Domain	dNO_3_
CARB (Caribbean)	Atlantic	Trade wind	0.13 ± 0.07
ETRA (Eastern tropical Atlantic)	Atlantic	Trade wind	0.56 ± 0.28
NATR (North Atlantic tropical gyral)	Atlantic	Trade wind	0.26 ± 0.25
SATL (South Atlantic gyral)	Atlantic	Trade wind	0.59 ± 0.56
WTRA (Western tropical Atlantic)	Atlantic	Trade wind	0.33 ± 0.12
MEDI (Mediterranean Sea)	Atlantic	Westerly	0.54 ± 0.58
NASE (Northeast Atlantic subtropical gyral)	Atlantic	Westerly	0.58 ± 0.33
NASW (Northwest Atlantic subtropical gyral)	Atlantic	Westerly	0.19 ± 0.10
ISSG (Indian South subtropical gyre)	Indian	Trade wind	0.31 ± 0.29
MONS (Indian monsoon gyre)	Indian	Trade wind	0.28 ± 0.08
ARAB (Northwest Arabian Sea upwelling)	Indian	Westerly	0.33 ± 0.09
ARCH (Archipelagic deep basins)	Pacific	Trade wind	0.17 ± 0.12
NPTG (North Pacific Tropical gyre)	Pacific	Trade wind	0.49 ± 0.45
PEQD (Pacific equatorial divergence)	Pacific	Trade wind	0.44 ± 0.33
PNEC (North Pacific equatorial counter)	Pacific	Trade wind	0.66 ± 0.29
SPSG (South Pacific gyre)	Pacific	Trade wind	0.51 ± 0.67
WARM (Western Pacific warm pool)	Pacific	Trade wind	0.10 ± 0.05
NPSW (Northwest Pacific subtropical)	Pacific	Westerly	0.12 ± 0.10
TASM (Tasman Sea)	Pacific	Westerly	0.96 ± 0.38

Mean ± standard deviation of nitrate diffusive fluxes (dNO_3,_ mmolN m^-2^ d^-1^_)_ calculated for the main open ocean biogeographical provinces defined in the Antarctic, Atlantic, Pacific and Indian oceans between 40° N–40° S by Longhurst^34^. Nitrate diffusive fluxes were calculated using the global model GM.

In general, a good correspondence was found between our model estimates and the limited number of studies that so far have quantified the magnitude of nitrate diffusive fluxes derived from observations of microstructure, tracer release experiments and high-resolution horizontal currents (Fig. 5 and Figure S2). However, a meaningful direct comparison between our mean estimates of nitrate diffusive fluxes with those derived from local observations, whose representativeness is limited in space and time, is very difficult to achieve. Turbulent mixing is highly intermittent in time and patchy in space due to its chaotic nature, and the multiple scales of variability of the forcing mechanisms. In the upper ocean, energy dissipation and mixing are linked to the exchanges of momentum and buoyancy with the atmosphere, which vary between seasons, day and night cycles and also following the synoptic weather patterns^35^. In the pycnocline, mixing also depends on highly unpredictable processes, such as the generation of shear by internal waves^36,37^. In coastal systems, tidal forcing can be a predictable source of mixing^38^, but it can also lead to complex dissipation patterns as a result of the production of non-linear internal waves^11,15^, and the interaction with wind-driven circulation^39^. Furthermore, the magnitude of nitrate fluxes is very sensitive to the different methodological choices made for the calculation, in particular to the depth-interval. Different depth criteria used for the estimates reported in Fig. 5 and Table 5 include the base of the euphotic zone, the base of the mixed layer, and also the nitracline. In fact, significant differences in nitrate diffusive fluxes computed in part of our dataset (the TRYNITROP cruise carried out in the subtropical Atlantic) were noticed depending on whether the base of the euphotic zone^28^ or the nitracline^18^ were chosen as depth-range for the calculation.

**Table 5:** Compilation of nitrate diffusive fluxes. Nitrate diffusive fluxes previously reported in the literature in several domains (D): tropical and subtropical regions (T) (40º N – 40ºS), Mediterranean Sea (M), shelf seas and upwelling regions (Te), and Polar regions (P).

D	Reference	Region	diffNO_3_ (mmolN m^−2^ d^−1^)
T	Lewis et al.^68^	Subtropical Northeast Atlantic	0.14 (0.002–0.89)
	Planas et al.^69^*	North, equatorial and south Atlantic	0.38 (0.00004–2.11)
	Dietze et al.^70^*	Subtropical North Atlantic	0.28 (0.14–0.41)
	Painter et al.^20^	Subtropical Northeast Atlantic	0.06 (0.04–0.09)
	Ledwell et al.^71^†	Sargasso Sea	0.1
	Sandel et al.^73^	Tropical Atlantic	2.4 (0.51–8.2)
	Carr et al.^74^	Equatorial Pacific	1.5 (0.01–3)
	Kaneko et al.^75^	Subtropical North Pacific	0.18 (0.02–0.35)
	Bouruet-Aubertot et al.^76^	Subtropical South Pacific	0.007 (0.003–0.011)
	Ellwood et al.^77^	Subtropical South Pacific	0.84 (0.02–1.65)
	Du et al.^78^	South China Sea	0.05 (0.00004–0.1)
M	Bonnet et al.^19^	Mediterranean Sea	0.002–0.013
Te	Horne et al.^77^	Georges Bank	0.12 (0.05–0.18)
	Hales et al.^78^	New England (Shelf Break)	0.22 (0.17–0.26)
	Cyr et al.^79^	Lower St. Lawrence Estuary	0.12–300
	Zhang et al.^80^	Florida Straits	42 (0.002–83.7)
	Forryan et al.^13^	Iceland Basin	0.13 (0.08–0.22)
	Martin et al.^81^	Porcupine Abyssal Plain	0.09 (0.05–0.16)
	Bendtsen & Richardson^82^	North Sea (Shelf Edge)	0.25 (0.1–2)
	Law et al.^83^ †	North Atlantic	1.8
	Rippeth et al.^84^	Irish Sea	1.5
	Williams et al.^85^	Irish Sea	0.31 (0.22–0.43)
	Sharples et al.^11^	Celtic Sea (Shelf Edge)	5.1 (1.3–9)
	Williams et al.^86^	Celtic Sea	12 (1.3–22)
	Sharples et al.^87^	English Channel	2 (0.8–3.2)
	Tweddle et al.^88^	Celtic Sea (Jones Bank)	26.4 (0.8–52)
	Schafstall et al.^89^	Mauritanian Upwelling Region	5.5 (1–10.1)
	Arcos-Pulido et al.^90^	Cape Ghir upwelling region	0.09
	Hales et al.^91^	Oregon Shelf	8.6
	Li et al.^92^	California current	1.3 (0–41.5)
	Sharples et al.^93^	New Zealand Shelf	12
P	Liu et al.^94^	China Sea (Shelf Edge)	0.22 (0.02–1.54)
	Sundfjord et al.^95^	Barents Sea	1.3 (0.15–2.4)
	Law et al.^96^ †	Antarctic Circumpolar Current	0.17
	Bourgault et al.^97^	Amundsen Gulf	0.5 (0.3–0.8)
	Randelhoff et al.^98^	Artic Ocean	0.5 (0.3–0.5)

The mean (and the range, between brackets) of estimates reported at each study are indicated. When the mean was not provided this value was calculated considering the published range. Publications reporting the dataset included in this analysis are not listed. When several frontiers for the calculation were used, the nitracline was choose. A molar mass of 62 gmol^−1^ was used for converting nitrate concentration from mg to mmol used in some of the references listed. * indicates estimates derived from acoustic Doppler and † from tracer release experiments.

A few studies have presented similar attempts to model nitrate availability in the surface waters by using different oceanographic predictors. In an early effort, Kamykowski and Zentara^40^ investigated the capability to predict nutrient concentrations from temperature or density using a global dataset. With satellite SST observations, Switzer et al.^41^ generated an index of nitrate availability in the surface waters of the global ocean. Steinhoff et al.^42^ proposed a proxy for nitrate in the North Atlantic Ocean, using multiple linear regression and observed nitrate and SST, and model-based MLD. Arteaga et al.^43^ developed a method for estimating global monthly mean surface nitrate by using local multiple linear regressions and including satellite SST and sCHL-a, and modelled MLD. Finally, Liang et al.^44^ used multivariate empirical orthogonal functions constructed from modeled nitrate, salinity, and potential temperature fields to build nitrate maps in the Southern Ocean. All these studies have assumed implicitly that the nitrate concentration alone is sufficient to characterize the variability in the supply of this limiting resource for phytoplankton growth. Instead, our study rather focuses on the rate at which nitrate is supplied to the euphotic zone by turbulent diffusion.

### Contribution of nitrate diffusion to primary production and export

We next investigated how our estimates fit in the nitrogen budget for tropical and subtropical regions, where most of our observations were collected. We first computed the spatial mean of nitrate diffusive fluxes between 40° N and 40° S using the global model. In order to limit our calculation to open ocean oligotrophic waters, we selected those regions where surface chlorophyll-a and nitrate diffusive fluxes were lower than 0.5 mg m^−3^ and 1 mmol m^−2^ d^−1^, respectively. Mean nitrate diffusive flux for this region (~ 20 Tmol N y^−1^) was comparable to the sum of global estimates of nitrogen fixation derived from models and observations (~ 11–14 Tmol N y^−1^^45–47^), fluvial inorganic nitrogen fluxes (0.05–0.71 Tmol N y^−1^)^48^ and atmospheric nitrogen deposition (~ 2.1 Tmol N y^−1^^45^) to the ocean. This figure was also comparable with an estimate of nitrate entrainment due to seasonal mixed-layer deepening between 40° N–40° S using climatological data (~ 12 Tmol N y^−1^, see Methods and Figure S3). These results indicate that, together with mixed-layer entrainment, nitrate diffusive flux represents a major source of new nitrogen into the euphotic zone in these regions. Assuming Redfield stoichiometry (C:N = 6.6), this flux may support at least 13–32% of the global export production of organic carbon, which is estimated to be 5–12 PgC y^−1^^49^.

The sum of our estimates of nitrate diffusion and nitrate entrainment for the region 40° N–40° S, and the global estimates of nitrogen fixation, fluvial nitrogen fluxes and atmospheric nitrogen deposition (~ 46–50 Tmol N y^−1^) represented ~ 37–40% (f-ratio = 0.37–0.40) of the regional mean net primary production derived from satellite data (183 ± 108 Tmol N y^−1^, see Methods). This is higher than previous estimates of the f-ratio in these regions, which according to several studies using sediment traps and modelling approaches fall within the range 0.1–0.2^2,4,50–52^. However, it is consistent with higher estimates of f-ratio derived for the Sargasso Sea from considering most identified physical and biological nitrogen supply mechanisms (see Table 2 in Lipschultz et al.^5^).

## Conclusions

Because nitrate availability is not necessarily correlated with nitrate concentration, it is crucial to quantify turbulent diffusion, traditionally considered one of the main mechanisms supplying new nitrogen to the phytoplankton cells inhabiting the surface layers of the ocean. By using a large dataset of microturbulence observations collected in contrasting marine environments and a multivariable fractional polynomial method, we built regional and global models that included a maximum of only three variables as predictors (surface temperature, SST; nitrate vertical gradient, grNO_3_, and surface chlorophyll-a, sCHL-a). The global model, which included the first two predictors, explained 50% of the variance in nitrate turbulent diffusion. Regional models for the Northwestern Mediterranean (including SST and sCHL-a) and for the Antarctic Peninsula (including SST) explained, respectively, 63% and 75% of the variance. In contrast, regional models for tropical and subtropical regions and for the Galician coastal upwelling (both including grNO_3_) explained, respectively, 27% and 49% of the variance. The global model applied to temperature and nitrate collected from databases provided the first large-scale map of nitrate supply into the euphotic zone through turbulent diffusion.

Given that chlorophyll is an estimator of phytoplankton biomass, which in turn depends on nutrient availability, including this variable as a predictor limits the application of these models for the study of the role that nitrate availability plays in controlling phytoplankton biomass and productivity. However, this approach can be very useful for constraining biogeochemical budgets. Mean nitrate diffusive flux (~ 20 Tmol N y^-1^) in oligotrophic tropical and subtropical regions was comparable to nitrate entrainment due to seasonal mixed-layer deepening between 40° N–40° S, and to the sum of global estimates of nitrogen fixation, fluvial fluxes and atmospheric deposition. This result confirms this process as one of the major mechanisms of new nitrogen supply into the surface oligotrophic ocean. The model presented here allows to quantify nitrate availability in the euphotic zone, globally and in selected regions, which is critical for understanding the export efficiency of organic carbon production, and its spatio-temporal variability, in response to natural and anthropogenic ocean change.

## Methods

### Sampling

During all cruises except COUPLING hydrographic properties and turbulent mixing were derived from a MSS microstructure profiler^53^. The MSS is equipped with a high-precision Conductivity-Temperature-Depth (CTD) probe, two microstructure shear sensors (type PNS06), and also a sensor to measure the horizontal acceleration of the profiler. The fluorometer included in the MSS profiler was calibrated with fluorometrically determined chlorophyll a concentrations ranging from 0.03 to 8.60 mg m^−3^ (Chl a = 2.255 × fluorescence – 0.527; R^2^ = 0.859, number of samples (ns) = 134), obtained during 12 cruises (see below). Measurements of dissipation rates of turbulent kinetic energy (ε) were conducted to the bottom, or to 243 ± 23 m over deep waters (Table 1). The MSS profiler was balanced to have negative buoyancy and a sinking velocity of ~ 0.4 to 0.7 m s^−1^. The frequency of data sampling was 1024 Hz. The sensitivity of the shear sensors was checked after each use. Data processing and calculation of dissipation rates of ε was carried out using the commercial software MSSpro^53^. During COUPLING turbulent mixing was derived from a TurboMAP microstructure profiler^54^. Data processing and calculation of dissipation rates of turbulent kinetic energy ε were carried out with the commercial software of the TurboMAP, as described in Sangrá et al.^55^.

Microstructure turbulence profiles used for computing nitrate fluxes at each station were always deployed successively. Sets include 2–11 in the tropical and subtropical regions, 6–94 in the Mediterranean, 2-402 in the Galician coastal upwelling, and 2–3 in the Antarctic Peninsula. Due to significant turbulence generation close to the ship, only the data below 5 m (HERCULES2, HERCULES3, DISTRAL, REIMAGE, STRAMIX, ASIMUTH, CHAOS, and NICANOR) and 10 m (CARPOS, TRYNITROP, COUPLING, MALASPINA, PERFILC, PERFILM, FAMOSO1, FAMOSO2, FAMOSO3) were considered reliable. Data were depth-averaged by calculating mean values within 1 m bins.

The squared buoyancy frequency (N^2^) was computed from the CTD profiles according to the equation:

$${N}^{2}=-\left(\frac{\mathrm{g}}{{\rho }_{w}}\right)\left(\frac{\partial \rho }{\partial z}\right)\left({s}^{-2}\right)$$

where g is the acceleration due to gravity (9.8 m s^−2^), *ρ*_*w*_ is a reference seawater density (1025 kg m^−3^), and *∂ρ/∂z* is the vertical potential density gradient. Vertical diffusivity (*K*_*z*_) was estimated as:

$${K}_{z}=\Gamma \frac{\varepsilon }{{N}^{2}} \left({m}^{2}{s}^{-1}\right)$$

where $\Gamma$ is the mixing efficiency, here considered as 0.2^56^ for all cruises except MALASPINA, TRYNITROP and CARPOS, which included stations with favorable conditions for double diffusion through salt-fingers. During these cruises vertical diffusivity including mechanical turbulence and the effect of salt-fingers mixing was calculated according to St. Laurent and Schmitt^57^ (see details in Fernández-Castro et al.^18^).

### Nitrate supply

Samples for the determination of nitrate (NO_3_) + nitrite (NO_2_) were collected from 5 ± 2 (Galician coastal upwelling), 7 ± 1 (Mediterranean), 11 ± 2 (Antarctic Peninsula) and 11 ± 2 (tropical and subtropical regions) different depths in rinsed polyethylene tubes and stored frozen at − 20 °C until analysis, according to standard methods using the automated colorimetric technique^58^. Analyses were performed on land except during the MALASPINA expedition where the samples were analyzed on board.

When nitrate concentrations were not available, during STRAMIX and one sampling during the NICANOR cruises, concentration values were obtained from a nitrate–density (σ_t_) relationship built by using all samples collected during CHAOS (ns = 624) and NICANOR (ns = 52), respectively. In CHAOS the nitrate-density relationship (NO_3_ = 11.93 × σ_t_ − 310.60; Adj-R^2^ = 0.92; p < 0.001) was valid for the density range between 25.9 and 27.2 kg m^−3^. During NICANOR the relationship showed a linear behavior (NO_3_ = 9.78 × σt − 256.38; Adj-R^2^ = 0.87; p < 0.001) for density ranging between 26.1 and 27.1 kg m^−3^^32^. Nitrate concentration profiles for PERFILM and PERFILC cruises were obtained from the World Ocean Atlas 2009 (WOA09,. http://www.nodc.noaa.gov/) as the closest available bin to the mean geographical location of each cruise (40.770° N–2.675° E and 43.865° N–2.173° W, respectively), and considering the mean values for the month when each cruise was conducted. The WOA09 was also used to obtain nitrate profiles at four stations during MALASPINA where samples for the determination of nutrients were not collected^18^.

Vertical diffusive fluxes of nitrate into the euphotic zone were calculated following the Fick’s law as:

$$Flux{NO}_{3}=\overline{{K }_{z}} \Delta {NO}_{3}$$

where $\Delta {NO}_{3}$ is the nitrate vertical gradient obtained by linear fitting of nitrate concentrations in the nitracline, determined as a region of approximately maximum and constant gradient, and $\overline{{K }_{z}}$ is the mean turbulent mixing in the same depth interval. In the Galician coastal upwelling, nitrate diffusive fluxes were estimated between 10 and 40 m depth using the same procedure.

The total nitrate supply in the Galician Rías was computed as the sum of nitrate vertical diffusion plus nitrate vertical advection due to coastal upwelling. A simplified estimate of nitrate supply through vertical advection due to upwelling was computed considering the Galician Rías as single boxes divided into two layers, the deeper one influenced by upwelled inflowing waters and the surface layer dominated by the outgoing flow. Assuming that the bottom layer volume is conservative and stationary, the vertical advective flux (Q_Z_, m^3^ s^−1^), would be equivalent to the incoming bottom flux (Q_B_, m^3^ s^−1^), computed as the product of the upwelling index (I_W_, m^3^ s^−1^ km^−1^) and the lengths of the mouth of the Rías (ca. 10–11.5 km). I_W_ was averaged by calculating the mean value over the three-day period before each cruise from wind data recorded by meteorological buoys located in Cabo Vilano (HERCULES, NICANOR) and Cabo Silleiro (DISTRAL-REIMAGE, ASIMUTH, CHAOS, STRAMIX, REIMAGE), or modeled by the Fleet Numerical Meteorology and Oceanography Center (FNMOC) model when buoy data were not available (http://www.indicedeafloramiento.ieo.es). Finally, the transport of nitrate into the euphotic zone through vertical advection was computed as:

$${NO}_{3}^{-} advective\;flux = \frac{{Q}_{Z}}{{A}_{basin}}{{[NO}_{3}^{-}]}_{ D}$$

where A_basin_ is the surface area of the Galician Rías, Q_Z_ is the vertical advective flux, and [NO_3_]_D_ is the mean nitrate concentration at the base of the euphotic layer. A_basin_ is 141 km^2^ for Ría de Pontevedra (ASIMUTH), 174 km^2^ for Ría de Vigo (CHAOS, ASIMUTH, DISTRAL-REIMAGE, STRAMIX), and 145 km^2^ for Ría de A Coruña (HERCULES, NICANOR).

Total nitrate supply included diffusive and advective vertical processes in the Galician costal upwelling, and only nitrate turbulent diffusion in the other regions. These results were only used to describe the magnitude of total nitrate supply in the four regions, whereas only nitrate diffusion was used as the response variable in the prediction models.

### Chlorophyll-a and primary production

Samples for the determination of chlorophyll-a were collected at four to eight depths during CARPOS (number of stations (n) = 8), TRYNITROP (n = 18), MALASPINA (n = 47), FAMOSO (n = 18), NICANOR (n = 13), HERCULESIII (n = 1), DISTRAL (n = 9), CHAOS (n = 2), and COUPLING (n = 9) cruises. 50–250 mL of seawater were filtered through 0.2 μm pore-size polycarbonate or Whatman GF/F (FAMOSO) filters which were later frozen at −20 °C until analysis. The fluorescence emitted by the chlorophyll-a was measured from pigments extracted in 90% acetone overnight. The fluorescence due to chlorophyll-a was measured using a Turner TD-700 fluorometer previously calibrated with pure chlorophyll-a. For those cruises where samples for the determination of chlorophyll-a were not collected, chlorophyll-a was derived from the calibrated fluorescence sensor included in the MSS profiler.

Samples for the determination of net primary production were collected at selected stations and several depths during TRINITROP (n = 18), MALAPINA (n = 37), FAMOSO (n = 19), NICANOR (n = 13), HERCULESIII (n = 1), DISTRAL (n = 8) and COUPLING (n = 3) cruises. During TRYNITROP, MALAPINA, NICANOR, HERCULESIII, and DISTRAL water samples from two to seven depths were collected for the determination of primary production with the ^14^C- uptake technique during on-deck incubations. For each depth, 75-mL (250-ml MALAPINA, NICANOR & HERCULESIII) polystyrene bottles (3 light and 1 dark bottles, or 2 light and 1 dark bottles for FAMOSO, NICANOR & HERCULESIII) were filled with seawater just before dawn and spiked with 2–15 μCi (50–100 μCi MALASPINA) of NaH^14^CO_3_. Samples were incubated during 2 h (DISTRAL), 24 h (MALASPINA, FAMOSO, NICANOR & HERCULESIII) or from dawn to dusk (TRYNITROP) in flow-through incubators provided with neutral density and blue (Lee Mist Blue) filters that simulated the PAR levels experienced by the phytoplankton in their natural location within the water column. The incubators were cooled with running seawater pumped from the surface or with water circulating through a refrigerator. At the end of the incubations, samples were filtered through 0.2 μm polycarbonate filters under low-vacuum pressure (< 100 mm Hg). Inorganic carbon on the filters was removed by exposing them to concentrated HCl fumes overnight. After removal of inorganic ^14^C, filters were placed into scintillation vials to which 4 mL of scintillation cocktail were added. The radioactivity on each sample was determined on scintillation counters which used an internal standard for quenching correction. Detailed methods for the experiments conducted during these cruises is included in Mouriño-Carballido et al.^28^, Marañón et al.^59^, Estrada et al.^60^, Moreira-Coello et al.^32^, and Cermeño et al.^29^.

During COUPLING primary production was measured using the ^13^C method^61^ at two sampling depths (surface and at the depth of the deep chlorophyll maximum). Water samples were transferred to 2 L polycarbonate bottles, and after addition of NaH^13^CO_3_ at about 10% of total inorganic carbon in the ambient water, the samples were incubated for about 12 h in a tank on deck. Initial and final particulate organic carbon, and particulate material used for isotope analysis were filtered through GF/F filters. They were frozen and stored at − 20 °C until analysis on land. Detailed methods are included in Teira et al.^30^.

For those experiments where incubations lasted less than 24 h, net primary production was computed assuming the ratio of phytoplankton respiration to gross photosynthesis (~ 20%; Geider^62^), and the number of light hours for each sampling date. Depth-integrated values were computed by trapezoidal integration considering the rates measured at the different depths.

### Model regression variables and statistical analysis

A Multivariable Fractional Polynomial (MFP) method was used to select the predictors of nitrate turbulent diffusion in the four contrasting environments (tropical and subtropical Atlantic Ocean, Northwestern Mediterranean Sea, Galician coastal upwelling ecosystem and Antarctic Peninsula), and for the complete dataset. Regression analyses commonly assume that relationships are linear between the predictors and the response variable, or assume a log-transform without assessing these assumptions. When addressed, nonlinear relationships are often based on generalized additive models or use standard polynomials. However, these models neither can be expressed as an explicit equation nor handle general nonlinearity, respectively. The MFP method solves these problems and determines whether a predictor is important for the model, and its functional form. The algorithm use fractional polynomials based on box-cox transformation^63^ of predictors. It uses the eight powers − 2, − 1, − 0.5, 0, 0.5, 1, 2 and 3 (with the 0 case corresponding to the natural log transform). A back-forward procedure is applied to include/exclude the terms of the model following the next steps: (I) backward elimination of predictors that are statistically insignificant, and (II) iterative examination of the scale of all continuous predictors. Therefore, we need two significance levels: α_1_, for the exclusion and inclusion of a predictor, and α_2_ for the determination of significance of fractional transformation of continuous predictors. Successive cycles improve the accuracy of the model until two cycles converge. Goodness of fit was assessed via quantile–quantile plots (Figure S1). All calculations were done using mfp package^33^ in R^64^.

### Global spatial distribution of nitrate turbulent diffusion

The large-scale spatial distribution of nitrate turbulent diffusion was computed by using the MFP model obtained by using the complete dataset except those stations collected in the Antarctic Peninsula, and including as predictors sea surface temperate and the vertical nitrate gradient across the nitracline derived from the WOA09. At each bin from WOA09 the vertical nitrate gradient was calculated as the maximal vertical gradient between 0 and 250 m. Regions where predictors data were outside the range covered by the dataset used to build the model were removed. Mean nitrate fluxes for most of the biogeographical provinces defined by Longhurst^34^ were calculated using the 3-variable model built with the complete dataset except those stations collected in the Antarctic Peninsula (GM). Mean nitrate diffusive flux between 40° N and 40° S was calculated using the 3-variable model GM, and only considering those regions where sCHL provided by the GlobColour Project from the European Space Agency (ESA, hermes.acri.fr) and nitrate diffusive fluxes were lower than 0.5 mg m^−3^ and 1 mmol m^−2^ d^−1^, respectively.

### Contribution of nitrate turbulent diffusion to net primary production in the 40° N–40° S region

Spatial mean total net primary production for the region between 40º N and 40º S was derived from satellite monthly means (period 1998–2012) by using the model proposed by Uitz et al.^65^, which was depth-integrated down to the base of the photic layer, computed from the algorithm proposed by Morel et al.^66^. In order to convert net carbon production to nitrogen units we assumed mean stoichiometry relationship for carbon and nitrogen proposed by Galbraith and Martiny^67^. Only those regions where sCHL and nitrate diffusive fluxes were lower than 0.5 mg m^−3^ and 1 mmol m^−2^ d^−1^, respectively, were considered.

### Nitrate entrainment due to seasonal mixed-layer deepening

The entrainment flux of nitrate into the mixed-layer was calculated from monthly climatological nitrate from the World Ocean Atlas 2013 as:

$${\mathbf{F}\mathbf{l}\mathbf{u}\mathbf{x}}_{\mathbf{N}\mathbf{O}3}^{\mathbf{e}\mathbf{n}\mathbf{t}\mathbf{r}\mathbf{a}\mathbf{i}\mathbf{n}\mathbf{m}\mathbf{e}\mathbf{n}\mathbf{t}}={\varvec{\Theta}}(\frac{\mathbf{d}\mathbf{M}\mathbf{L}\mathbf{D}}{\mathbf{d}\mathbf{t}})({\mathbf{N}\mathbf{O}}_{3}^{\mathbf{t}\mathbf{h}}-{\mathbf{N}\mathbf{O}}_{3}^{\mathbf{m}\mathbf{l}})$$

where dMLD/dt is the rate of deepening of the mixed layer between two consecutive months (*i* and *i* + 1, with *i* = 1,…,12); ${{\varvec{N}}{\varvec{O}}}_{3}^{{\varvec{m}}{\varvec{l}}}$ and ${{\varvec{N}}{\varvec{O}}}_{3}^{{\varvec{t}}{\varvec{h}}}$ are the mean nitrate concentrations on month *i* in the first mixed layer (i.e. between the surface and MLD^i^) and in the thermocline (between MLD^i^ and MLD^i+1^), respectively, and ${\varvec{\Theta}}$ is the Heaviside function, being ${\varvec{\Theta}}$ = dMLD/dt when dMLD/dt > 0 and ${\varvec{\Theta}}$ = 0 otherwise. The entrainment fluxes were calculated for each gridpoint of the WOA13 and for every pair of months and then time-averaged to obtain an annual mean estimate. Mixed layer depths where calculated from the monthly profiles of potential density as the depth were potential density exceeded the near-surface value by 0.1 kg m^-3^.

## Supplementary Information

Supplementary Information.

## References

[1] Capone DG, Radu P, Flood B, Nealson KH (2006). Follow the nitrogen. Science.

[2] Dugdale RC, Goering JJ, Apr N (1967). Uptake of new and regenerated forms of nitrogen in primary productivity. Limnol. Oceanogr..

[3] Lewis MR, Harrison WG, Oakey NS, Hebert D, Platt T (1986). Vertical nitrate fluxes in the oligotrophic ocean. Science.

[4] Eppley RW, Peterson BJ (1979). Particulate organic matter flux and planktonic new production in the deep ocean. Nature.

[5] Lipschultz F, Bates NR, Carlson CA, Hansell DA (2002). New production in the Sargasso sea: History and current status. Global Biogeochem. Cycles.

[6] Bendtsen J, Richardson K (2018). Turbulence measurements suggest high rates of new production over the shelf edge in the north-eastern North Sea during summer. Biogeosciences.

[7] Rippeth TP (2005). Mixing in seasonally stratified shelf seas: A shifting paradigm. Philos. Trans. R Soc. A Math. Phys. Eng. Sci..

[8] Thorpe SA (2007). An Introduction to Ocean Turbulence.

[9] Capone DG (2005). Nitrogen fixation by *Trichodesmium* spp.: An important source of new nitrogen to the tropical and subtropical North Atlantic Ocean. Global Biogeochem. Cycles.

[10] Hibiya T, Nagasawa M, Niwa Y (2007). Latitudinal dependence of diapycnal diffusivity in the thermocline observed using a microstructure profiler. Geophys. Res. Lett..

[11] Sharples J (2007). Spring-neap modulation of internal tide mixing and vertical nitrate fluxes at a shelf edge in summer. Limnol. Oceanogr..

[12] Cuypers Y, Bouruet-Aubertot P, Marec C, Fuda J-L (2012). Characterization of turbulence from a fine-scale parameterization and microstructure measurements in the Mediterranean Sea during the BOUM experiment. Biogeosciences.

[13] Forryan A (2012). Turbulent nutrient fluxes in the Iceland Basin. Deep Sea Res Part I Oceanogr. Res. Pap..

[14] Fernández-Castro B (2014). Microstructure turbulence and diffusivity parameterization in the tropical and subtropical Atlantic, Pacific and Indian Oceans during the Malaspina 2010 expedition. Deep. Res. Part.

[15] Villamaña M (2017). Role of internal waves on mixing, nutrient supply and phytoplankton community structure during spring and neap tides in the upwelling ecosystem of Ría de Vigo (NW Iberian Peninsula). Limnol. Oceanogr..

[16] Bouruet-Aubertot P (2018). Longitudinal contrast in turbulence along a ∼ 19°S section in the Pacific and its consequences for biogeochemical fluxes. Biogeosciences.

[17] Tuerena RE (2019). Internal tides drive nutrient fluxes into the deep chlorophyll maximum over mid-ocean ridges. Global Biogeochem. Cycles.

[18] Fernández-Castro B (2015). Importance of salt fingering for new nitrogen supply in the oligotrophic ocean. Nat. Commun..

[19] Bonnet S, Grosso O, Moutin T (2011). Planktonic dinitrogen fixation along a longitudinal gradient across the Mediterranean Sea during the stratified period (BOUM cruise). Biogeosciences.

[20] Painter SC, Patey MD, Forryan A, Torres-Valdes S (2013). Evaluating the balance between vertical diffusive nitrate supply and nitrogen fixation with reference to nitrate uptake in the eastern subtropical North Atlantic Ocean. J. Geophys. Res. Ocean..

[21] Caffin M (2018). N2 fixation as a dominant new N source in the western tropical South Pacific Ocean (OUTPACE cruise). Biogeosciences.

[22] Agawin NSR, Duarte CM, Agusti S (2000). Nutrient and temperature control of the contribution of picoplankton to phytoplankton biomass and production. Limnol. Oceanogr..

[23] Flombaum P (2013). Present and future global distributions of the marine cyanobacteria prochlorococcus and synechococcus. Proc. Natl. Acad. Sci..

[24] Mouriño-Carballido B (2016). Nutrient supply controls picoplankton community structure during three contrasting seasons in the northwestern Mediterranean Sea. Mar. Ecol. Prog. Ser..

[25] Otero-Ferrer JL (2018). Factors controlling the community structure of picoplankton in contrasting marine environments. Biogeosciences.

[26] Álvarez-Salgado XA, Gago J, Míguez, B. M., Gilcoto, M. & Pérez, F. F. (2000). Surface waters of the NW Iberian margin: Upwelling on the shelf versus outwelling of upwelled waters from the Rías Baixas. Estuar Coast Shelf Sci..

[27] Aranguren-Gassis M (2011). Production and respiration control the marine microbial metabolic balance in the eastern north atlantic subtropical gyre. Deep Res. Part I Oceanogr. Res. Pap..

[28] Mouriño-Carballido B (2011). Importance of N 2 fixation vs nitrate eddy diffusion along a latitudinal transect in the Atlantic Ocean. Limnol. Oceanogr..

[29] Cermeño P (2016). Marine primary productivity is driven by a selection effect. Front. Mar. Sci..

[30] Teira E (2012). Primary production and bacterial carbon metabolism around South Shetland Islands in the Southern Ocean. Deep Res. Part I Oceanogr. Res. Pap..

[31] Díaz PA (2019). Fine scale physical-biological interactions during a shift from relaxation to upwelling with a focus on Dinophysis acuminata and its potential ciliate prey. Prog. Oceanogr..

[32] Moreira-Coello V (2017). Biological N2 Fixation in the upwelling region off NW Iberia: Magnitude, relevance, and players. Front. Mar. Sci..

[33] Zhang Z (2016). Multivariable fractional polynomial method for regression model. Ann. Transl. Med..

[34] Longhurst AR (2007). Ecological Geography of the Sea.

[35] Moum, J. N. & Smyth, W. D. Mixing processes. *Encycl. Ocean Sci.* **6**, 3093–3100. 10.1016/B978-0-12-409548-9.11573-8 (2001).

[36] Rippeth TP, Palmer MR, Simpson JH, Fisher NR, Sharples J (2005). Thermocline mixing in summer stratified continental shelf seas. Geophys. Res. Lett..

[37] Nash JD, Kelly SM, Shroyer EL, Moum JN, Duda TF (2012). The unpredictable nature of internal tides on continental shelves. J. Phys. Oceanogr..

[38] Simpson JH, Brown J, Matthews J, Allen G (1990). Tidal straining, density currents, and stirring in the control of estuarine stratification. Estuaries.

[39] Fernández-Castro B (2018). Modulation of the semidiurnal cycle of turbulent dissipation by wind-driven upwelling in a coastal embayment. J. Geophys. Res. Ocean..

[40] Kamykowski D, Zentara SJ (1986). Predicting plant nutrient concentrations from temperature and sigma-t in the upper kilometer of the world ocean. Deep Sea Res..

[41] Switzer AC (2003). Mapping nitrate in the global ocean using remotely sensed sea surface temperature. J. Geophys. Res..

[42] Steinhoff T (2010). Estimating mixed layer nitrate in the North Atlantic Ocean. Biogeosciences.

[43] Arteaga L, Pahlow M, Oschlies A (2015). Global monthly sea surface nitrate fields estimated from remotely sensed sea surface temperature, chlorophyll, and modeled mixed layer depth. Geophys. Res. Lett..

[44] Liang YC, Mazloff MR, Rosso I, Fang SW, Yu JY (2018). A multivariate empirical orthogonal function method to construct nitrate maps in the Southern Ocean. J. Atmos. Ocean. Technol..

[45] Jickells TD (2017). A reevaluation of the magnitude and impacts of anthropogenic atmospheric nitrogen inputs on the ocean. Global Biogeochem. Cycles.

[46] Tang W (2019). Revisiting the distribution of oceanic N2 fixation and estimating diazotrophic contribution to marine production. Nat. Commun..

[47] Wang W, Moore JK, Martiny AC, François W (2019). Convergent estimates of marine nitrogen fixation. Nature.

[48] Izett JG, Fennel K (2018). Estimating the cross-shelf export of riverine materials: Part 2. Estimates of global freshwater and nutrient export. Global Biogeochem. Cycles.

[49] Siegel DA (2014). Global assessment of ocean carbon export by combining satellite observations and food-web models. Global Biogeochem. Cycles.

[50] Dunne JP, Armstrong RA, Gnanadesikan A, Sarmiento JL (2005). Empirical and mechanistic models for the particle export ratio. Glob. Biogeochem. Cycles.

[51] Laws EA, D’Sa E, Naik P (2011). Simple equations to estimate ratios of new or export production to total production from satellite-derived estimates of sea surface temperature and primary production. Limnol. Oceanogr. Methods.

[52] Yool A, Martin AP, Fernández C, Clark DR (2007). The significance of nitrification for oceanic new production. Nature.

[53] Prandke H, Stips A (1998). Test measurements with an operational microstructure-turbulence profiler: Detection limit of dissipation rates. Aquat. Sci..

[54] Wolk F, Yamazaki H, Seuront L, Lueck RG (2002). A new free-fall profiler for measuring biophysical microstructure. J. Atmos. Ocean. Technol..

[55] Sangrà P (2014). Coupling between upper ocean layer variability and size-fractionated phytoplankton in a non-nutrient-limited environment. Mar. Ecol. Prog. Ser..

[56] Osborn TR (1980). Estimates of the local rate of vertical diffusion from dissipation meassurements. J. Phys. Oceanogr..

[57] St. Laurent, L. C. & Schmitt, R. W. (1999). The contribution of salt fingers to vertical mixing in the North Atlantic Tracer Release Experiment. J. Phys. Oceanogr..

[58] Hansen, H. P. & Koroleff, F. Determination of nutrients. in *Methods of Seawater Analysis* (eds. Grasshoff, K., Kremling, K. & Ehrhardt, M.) 159–228 (Wiley-VCH Verlag GmbH, 1999).. 10.1002/9783527613984

[59] Marañón E (2016). Coccolithophore calcification is independent of carbonate chemistry in the tropical ocean. Limnol. Oceanogr..

[60] Estrada M (2014). Seasonal and mesoscale variability of primary production in the deep winter-mixing region of the NW Mediterranean. Deep. Res. Part I Oceanogr. Res. Pap..

[61] Hama T (1983). Measurement of photosynthetic production of a marine phytoplankton population using a stable 13C isotope. Mar. Biol..

[62] Geider, R. J. Respiration: Taxation Without Representation? in *Primary Productivity and Biogeochemical Cycles in the Sea* 333–360 (Springer US, 1992).. 10.1007/978-1-4899-0762-2_19

[63] Box GEP, Cox DR (1964). An Analysis of Transformations. J. R. Stat. Soc. Ser. B.

[64] R Core Team. R: A Language and Environment for Statistical Computing. (2015).

[65] Uitz J, Huot Y, Bruyant F, Babin M, Claustre H (2008). Relating phytoplankton photophysiological properties to community structure on large scales. Limnol. Oceanogr..

[66] Morel A (2007). Examining the consistency of products derived from various ocean color sensors in open ocean (Case 1) waters in the perspective of a multi-sensor approach. Remote Sens. Environ..

[67] Galbraith ED, Martiny AC (2015). A simple nutrient-dependence mechanism for predicting the stoichiometry of marine ecosystems. Proc. Natl. Acad. Sci..

[68] Lewis MR, Hebert D, Harrison WG, Plat TS (1986). Vertical nitrate fluxes in the oligotrophic ocean. Science.

[69] Planas D, Agustí S, Duarte CM, Granata TC, Merino M (1999). Nitrate uptake and diffusive nitrate supply in the Central Atlantic. Limnol. Oceanogr..

[70] Dietze H, Oschlies A, Kahler P (2004). Internal-wave-induced and double-diffusive nutrient fluxes to the nutrient-consuming surface layer in the oligotrophic subtropical North Atlantic. Ocean Dyn..

[71] Ledwell JR, McGillicuddy DJ, Anderson LA (2008). Nutrient flux into an intense deep chlorophyll layer in a mode-water eddy. Deep Sea Res Part Top. Stud. Oceanogr..

[72] Sandel V (2015). Nitrogen fuelling of the pelagic food web of the Tropical Atlantic. PLoS ONE.

[73] Carr M-E, Lewis MR, Kelley D, Jones B (1995). A physical estimate of new production in the equatorial Pacific along 150°W. Limnol. Oceanogr..

[74] Kaneko H, Yasuda I, Komatsu K, Itoh S (2013). Observations of vertical turbulent nitrate flux across the Kuroshio. Geophys. Res. Lett..

[75] Ellwood MJ (2018). Insights Into the biogeochemical cycling of iron, nitrate, and phosphate across a 5,300 km south pacific zonal section (153°E–150°W). Global Biogeochem. Cycles.

[76] Du C, Liu Z, Kao SJ, Dai M (2017). Diapycnal fluxes of nutrients in an oligotrophic oceanic regime: The South China Sea. Geophys. Res. Lett..

[77] Horne EPW, Loder JW, Naimief CE, Oakey NS (1996). Turbulence dissipation rates and nitrate supply in the upper water column on Georges Bank. Deep. Res. Part II Top. Stud. Oceanogr..

[78] Hales B, Hebert D, Marra J (2009). Turbulent supply of nutrients to phytoplankton at the New England shelf break front. J. Geophys. Res. Ocean..

[79] Cyr F, Bourgault D, Galbraith PS, Gosselin M (2015). Turbulent nitrate fluxes in the Lower St. Lawrence Estuary. Canada. J. Geophys. Res. C Ocean..

[80] Zhang JZ, Baringer MO, Fischer CJ, Hooper VJA (2017). An estimate of diapycnal nutrient fluxes to the euphotic zone in the Florida Straits. Sci. Rep..

[81] Martin AP (2010). The supply of nutrients due to vertical turbulent mixing: A study at the Porcupine Abyssal Plain study site in the northeast Atlantic. Deep. Res. Part II Top. Stud. Oceanogr..

[82] Bendtsen J, Richardson K (2018). Turbulence measurements suggest high rates of new production over the shelf edge in the northeastern North Sea during summer. Biogeosciences.

[83] Law CS (2001). A Lagrangian SF 6 tracer study of an anticyclonic eddy in the North Atlantic: Patch evolution, vertical mixing and nutrient supply to the mixed layer. Deep. Res. Part II Top. Stud. Oceanogr..

[84] Rippeth TP, Wiles P, Palmer MR, Sharples J, Tweddle J (2009). The diapcynal nutrient flux and shear-induced diapcynal mixing in the seasonally stratified western Irish Sea. Cont. Shelf Res..

[85] Williams C, Sharples J, Green M, Mahaffey C, Rippeth T (2013). The maintenance of the subsurface chlorophyll maximum in the stratified western Irish Sea. Limnol. Oceanogr. Fluids Environ..

[86] Williams C, Sharples J, Mahaffey C, Rippeth T (2013). Wind-driven nutrient pulses to the subsurface chlorophyll maximum in seasonally stratified shelf seas. Geophys. Res. Lett..

[87] Sharples J (2001). Phytoplankton distribution and survival in the thermocline. Limnol. Oceanogr..

[88] Tweddle JF, Sharples J, Palmer MR, Davidson K, McNeill S (2013). Enhanced nutrient fluxes at the shelf sea seasonal thermocline caused by stratified flow over a bank. Prog. Oceanogr..

[89] Schafstall J, Dengler M, Brandt P, Bange H (2010). Tidal-induced mixing and diapycnal nutrient fluxes in the Mauritanian upwelling region. J. Geophys. Res..

[90] Arcos-Pulido M (2014). Diapycnal nutrient fluxes on the northern boundary of Cape Ghir upwelling region. Deep. Res. Part I Oceanogr. Res. Pap..

[91] Hales B (2005). Irreversible nitrate fluxes due to turbulent mixing in a coastal upwelling system. J. Geophys. Res..

[92] Li QP, Franks PJS, Ohman MD, Landry MR (2012). Enhanced nitrate fluxes and biological processes at a frontal zone in the southern California current system. J. Plankton Res..

[93] Sharples J, Moore CM, Abraham R (2001). Internal tide dissipation, mixing, and vertical nitrate flux. J. Geophys. Res..

[94] Liu X (2013). Variability in nitrogen sources for new production in the vicinity of the shelf edge of the East China Sea in summer. Cont. Shelf Res..

[95] Sundfjord A, Fer I, Kasajima Y, Svendsen H (2007). Observations of turbulent mixing and hydrography in the marginal ice zone of the Barents Sea. J. Geophys. Res. Ocean..

[96] Law CS, Abraham ER, Watson AJ, Liddicoat MI (2003). Vertical eddy diffusion and nutrient supply to the surface mixed layer of the Antarctic Circumpolar Current. J. Geophys. Res..

[97] Bourgault D (2011). Turbulent nitrate fluxes in the Amundsen Gulf during ice-covered conditions. Geophys. Res. Lett..

[98] Randelhoff A, Ilker F, Sundfjord A, Tremblay J-E, Reigstad M (2016). Vertical fluxes of nitrate in the seasonal nitracline of the Atlantic sector of the Arctic Ocean. J. Geophys. Res. Ocean.

