# Supplementary material for: Magnitude of nitrate turbulent diffusion in contrasting marine environments
Source: Sci Rep. 2021 Sep 22;11:18804. doi: 10.1038/s41598-021-97731-4 (PMC8458521; doi:10.1038/s41598-021-97731-4)
Supplement: Supplementary file 1 — Supplementary Information. [file 41598_2021_97731_MOESM1_ESM.docx]

Magnitude of nitrate turbulent diffusion in contrasting marine environments

**Beatriz Mouriño-Carballido^1^, José Luis Otero Ferrer^1^, Bieito Fernández-Castro^2^, Emilio Marañón^1^, Mariña Blazquez Maseda^1^, Borja Aguiar-González^3, 4^, Paloma Chouciño^1^, Rocío Graña^5^, Víctor Moreira-Coello^1^, Marina Villamaña^1^**

^1^ Departamento de Bioloxía e Ecoloxía Animal, Universidade de Vigo, Vigo, Spain.

^2^ Ocean and Earth Science, University of Southampton, National Oceanography Centre, SO14 3ZH, Southampton, UK

^3^ Departamento de Física, Facultad de Ciencias del Mar, Universidad de Las Palmas de Gran Canaria, 35017 Las Palmas, Spain.

^4^ School of Marine Science and Policy, College of Earth, Ocean and Environment, University of Delaware, Newark, Delaware, USA.

^5^ Instituto Español de Oceanografía, Xixón, Spain.

Corresponding author: Beatriz Mouriño-Carballido ([bmourino@uvigo.es)](mailto:email@address.edu))


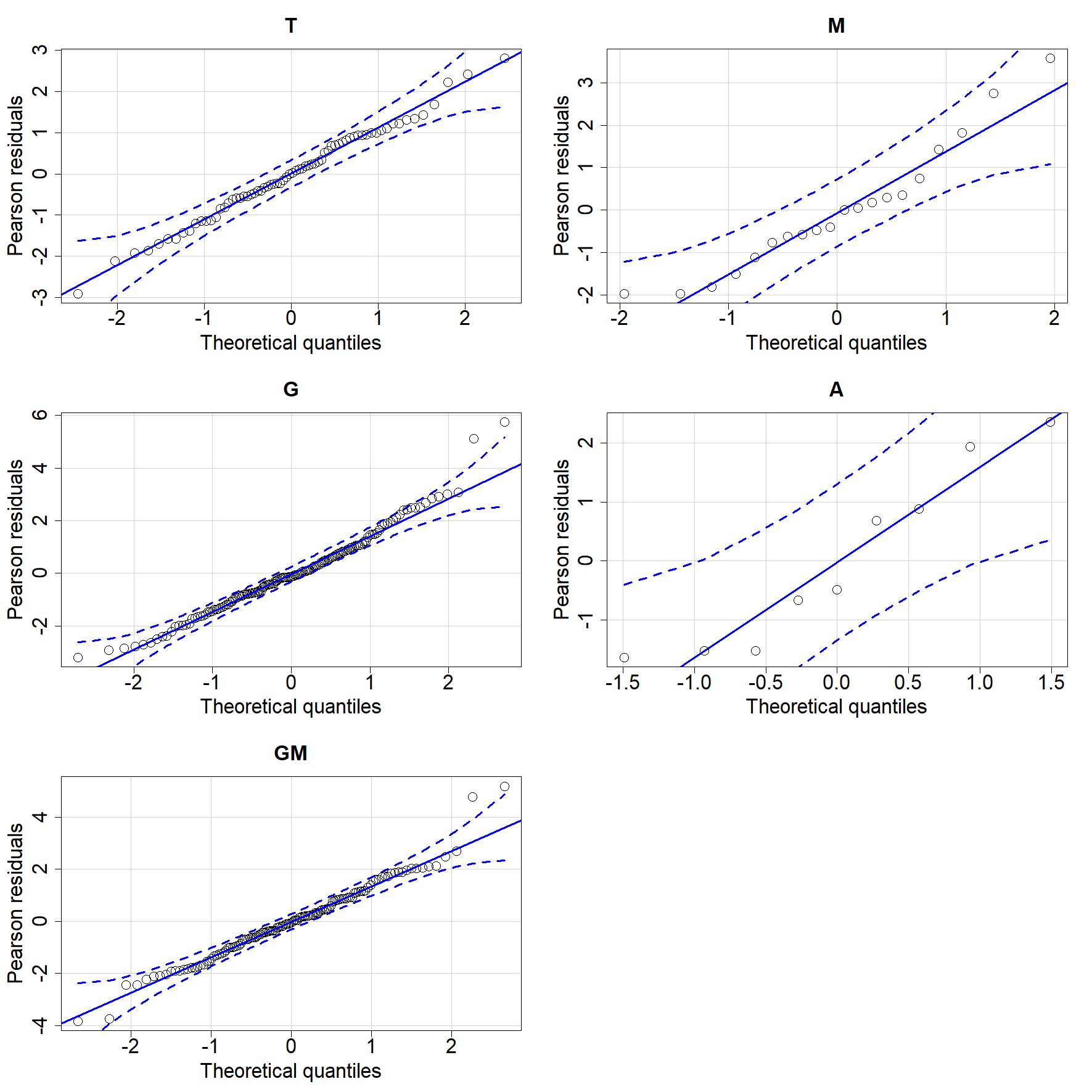


**Figure S1.** Quantile-Quantile (QQ) plots. Plots were obtained by the MFP method in each domain: tropical and subtropical regions (T), Mediterranean Sea (M), Galician coastal upwelling (G), Antarctic Peninsula (A), and using the complete dataset except those stations collected in the Antarctic Peninsula (GM). The y-axes represent the Pearson residuals and the x-axes the theoretical quantiles. Solid blue lines indicate the theoretical quantile of the models and blue dashed lines the 95% confidence intervals.

**
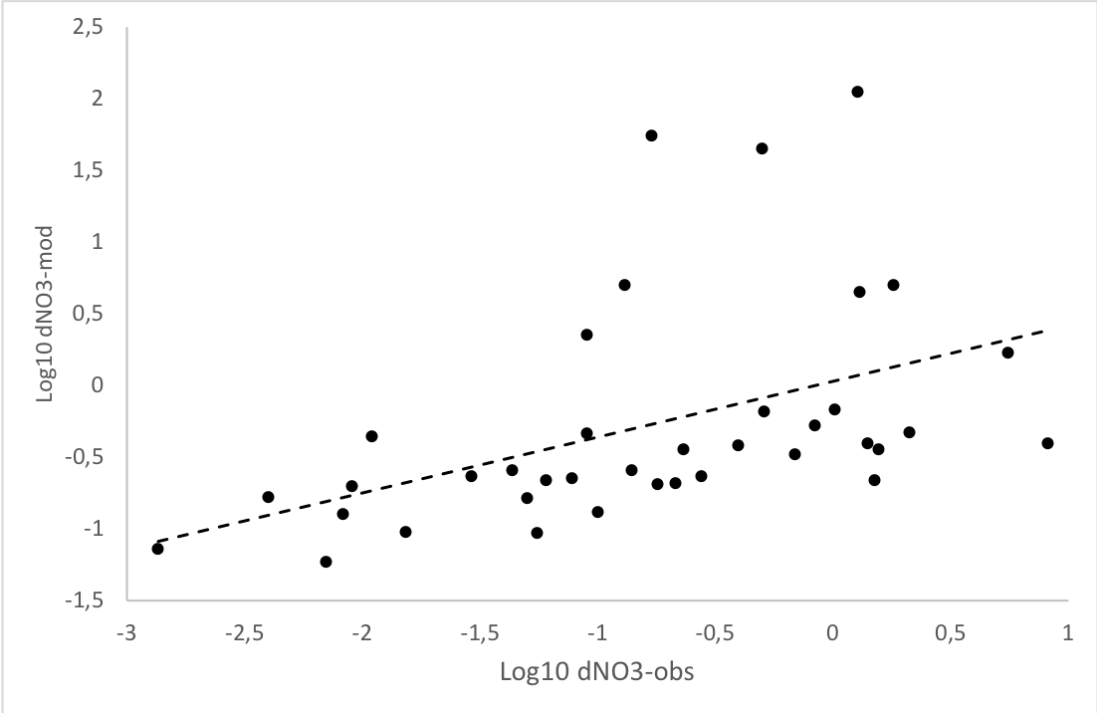
**

**Figure S2.** Comparison of observations versus model predicted values. Nitrate diffusive flux derived from observations (Log10 dNO3-obs, see Table 5) versus nitrate diffusive flux derived from the global model GM (Log10 dNO3-mod) for the same geographical locations. The dashed line represents the statistically significant relationship (y=0.39x+0.028; r^2^ = 0.21; p < 0.01) calculated using all data except the lowest two values reported by Planas et al. ^1^ in the North Atlantic (3.75-4.85 x 10^-5^ mmolN m^-2^ d^-1^) by using indirect methods based on acoustic Doppler.


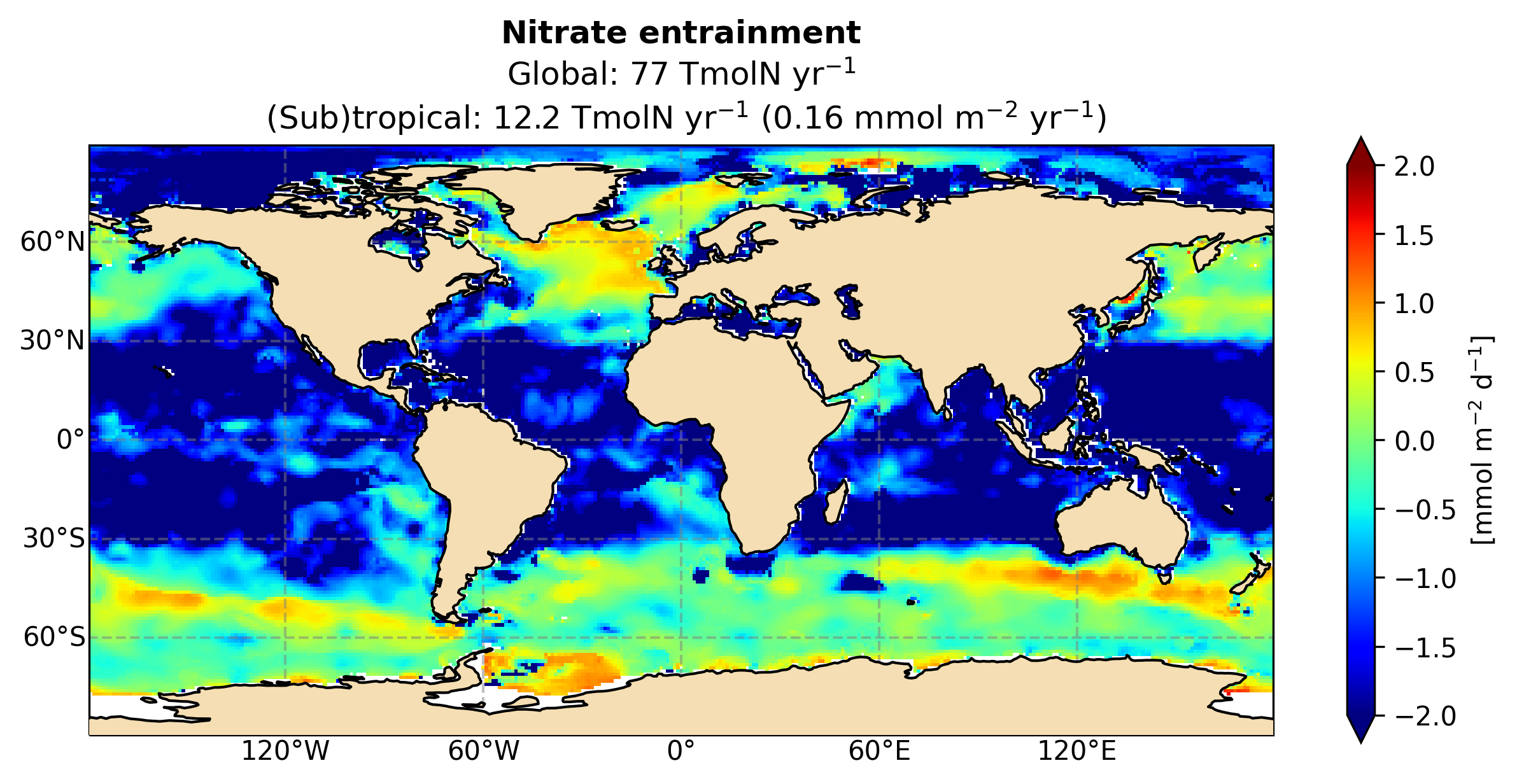


Figure S3. Mean annual nitrate entrainment flux into the surface layer due to seasonal mixed-layer deepening computed with monthly nitrate profiles and mixed-layer depth values derived from the World Ocean Atlas 2013. Global and regional areal integrals for the (sub)tropical ocean (between 40°S and 40°N) are reported. Figure was generated by using Python (<https://www.python.org>, version Python 3.9.6).

References

1. Planas, D., Agustí, S., Duarte, C. M., Granata, T. C. & Merino, M. Nitrate uptake and diffusive nitrate supply in the Central Atlantic. *Limnol. Oceanogr.* **44**, 116–126 (1999).
